# Natural transformation of the filamentous cyanobacterium *Phormidium lacuna*

**DOI:** 10.1371/journal.pone.0234440

**Published:** 2020-06-12

**Authors:** Fabian Nies, Marion Mielke, Janko Pochert, Tilman Lamparter

**Affiliations:** Botanical Institute, Karlsruhe Institute of Technology (KIT), Karlsruhe, Germany; INRA, FRANCE

## Abstract

Research for biotechnological applications of cyanobacteria focuses on synthetic pathways and bioreactor design, while little effort is devoted to introduce new, promising organisms in the field. Applications are most often based on recombinant work, and the establishment of transformation can be a risky, time-consuming procedure. In this work we demonstrate the natural transformation of the filamentous cyanobacterium *Phormidium lacuna* and insertion of a selection marker into the genome by homologous recombination. This is the first example for natural transformation filamentous non-heterocystous cyanobacterium. We found that *Phormidium lacuna* is polyploid, each cell has about 20–90 chromosomes. Transformed filaments were resistant against up to 14 mg/ml of kanamycin. Formerly, natural transformation in cyanobacteria has been considered a rare and exclusive feature of a few unicellular species. Our finding suggests that natural competence is more distributed among cyanobacteria than previously thought. This is supported by bioinformatic analyses which show that all protein factors for natural transformation are present in the majority of the analyzed cyanobacteria.

## Introduction

Biotechnology oriented research with cyanobacteria ranges from the production of low-cost material like bulk chemicals or biofuels [1] to high-value compounds like pharmaceutics [2]. Advancements in cyanobacterial biotechnology are based on continued optimization of photobioreactors, the introduction and improvement of metabolic pathways by recombinant DNA technology, and the search for suitable organisms [3, 4]. The establishment of protocols for gene transfer can be challenging for new cyanobacteria because of barriers like extracellular materials and nucleases (reviewed in [5]). There are three common methods for gene transfer into cyanobacteria: electroporation, conjugation, and natural transformation (NT). For NT, cells have to be in a physiological state, termed natural competence (NC), in which the recipient cell is able to actively transport DNA into the cytoplasm. Protocols for NT are generally simple and straight forward [6], but only few naturally competent cyanobacteria (NCC) are known: diverse *Synechococcus* [7, 8] and *Synechocystis* [9, 10] strains, *Microcystis aeruginosa* PCC 7806 [11] and *Thermosynechococcus elongatus* BP-1 [12]. These cyanobacteria have a unicellular lifestyle, and NT was frequently described as a unique feature of few unicellular cyanobacteria [6, 13, 14]. There is also one report about NT of *Nostoc muscorum* [15], which belongs to the filamentous cyanobacteria with heterocysts, but no report about other filamentous cyanobacteria.

DNA uptake in natural transformation is dependent on Pil proteins of type IV pili. Furthermore, the competence proteins ComEA, ComEC, and ComF as well as the DNA processing protein DprA and the DNA recombination and repair protein RecA are essential [16–18]. For the cyanobacterium *Synechocystis* sp. PCC 6803, *comEA*, *comF*, *pilA1*, *pilB1*, *pilD*, *pilM*, *pilN*, *pilO*, *pilQ*, and *pilT1* knockout mutants are deficient in NT [19–22]. The role of these proteins (among others) during natural transformation was recently also demonstrated in *Synechococcus elongatus* PCC 7942 by a transposon mutagenesis approach [23]. In recent surveys, homologs of these and other competence related genes were found in many cyanobacterial genomes [23, 24] but there is so far no experimental evidence for NT in a novel species since more than a decade.

During transformation, the introduced DNA can either be integrated into the genome by homologous recombination or must be on a self-replicating plasmid. For integration into the genome it must be considered that cyanobacteria might possess multiple chromosomes per cell [25–29]. Isolation of homozygous transformants can be achieved by selection on increasing antibiotic concentrations. Until complete segregation of selection marker is achieved, the transformants may be unstable and the integrated sequences can be lost again under nonselective conditions [30, 31].

In this work we established an NT protocol for *Phormidium lacuna*, a filamentous non-heterocystous cyanobacterium. Our workgroup has isolated several strains of this newly described species from marine rockpools on Helgoland, North Sea, Germany, and on Giglio, Mediterranean, Italy, and the genome of strain HE10JO was sequenced [32]. The genus was characterized as *Phormidium* based on sequences of 16S rRNA and of core proteins [32]. Among the different isolated strains, 16S RNA sequences are identical. We characterized *Phormidium lacuna* as a promising candidate for biotechnological applications because it is tolerant against differing salt concentrations, temperatures up to 50 °C, and strong light. *Phormidium lacuna* is cultivable on agar medium, in liquid culture, and in stirring tank photobioreactor [32]. On agar medium the filaments show twitching motility along their longitudinal axes, as other filamentous non-heterocystous cyanobacteria, e.g. *Oscillatoria salina* [33]. This report is to our knowledge the first report of a gene transfer for the genus *Phormidium* and the first report of NT for a filamentous cyanobacterium without heterocysts. *Phormidium lacuna* was transformed by the integration of the kanamycin (Km) resistance cassette (*kanR*) into the genome via homologous recombination. Clones were selected by Km resistance and integration into genome was validated by PCR. During clone validation it was found that *Phormidium lacuna* is polyploid. This was confirmed by a DAPI fluorescence assay. By comprehensive BLAST analysis based on sequences of essential proteins for natural transformation (natural transformation factors, NTFs) we predict that a large fraction of cyanobacteria might be naturally transformable.

## Material and methods

### Species and strains

*Phormidium lacuna* strains used in this study, HE10JO and HE10DO, were collected from marine rockpools in Helgoland, North Sea, as described [32]. The genome of strain HE10JO is sequenced [32]. The 16S rRNA sequence of HE10JO and HE10DO is identical while the phytochrome sequence differs at one position between the two strains [32]. We assume that both genomes are almost identical; also the sc_7_37 sequence is identical. *Synechocystis* sp. PCC 6803 was obtained from Annegret Wilde [34].

### Plasmids for transformation

The present transformation vectors contain a homologous sequence that is interrupted by a resistance cassette. The homologous sequences are based on a *Phormidium lacuna* open reading frame sequence sc_7_37, which is the 37^th^ open reading frame of DNA scaffold 7. sc_7_37 sequence encodes for a protein (Refseq ID: WP_087706519) that is annotated as hypothetical protein and which has close BLAST homologs that are annotated as hydrogenase. The coding sequences of both strains, HE10DO and HE10JO, are identical. An 1138 bp and a 2167 bp product were generated by PCR using Q5 polymerase (NEB, Ipswich, MA, USA) and genomic DNA of *Phormidium lacuna* as PCR template. The primer pairs were T256 / T257 for the short sequence and F114 / F115 for the long sequence, primers are listed in Table 1. The short and long sequences were integrated into pGEM-T by TA cloning (Promega, Madison, WI, USA). pGEM-T plasmids are not propagated in cyanobacteria due to incompatible origin of replication [35].

**Table 1:** List of PCR primers.

Primer name	Sequence
**F5**	CAACAAGCTAGCGTTTGCGAGGCTAAAGGCG
**F6**	CAACAATCTAGAGGTTCCCACTCCCAAAGC
**F13**	CAACAATCTAGACTCGTATGTTGTGTGGAATTG
**F14**	CAACAAGCTAGCCAAGTCAGCGTAATGCTCTG
**F25**	GGTCTAGGTGAGGCAATCC
**F28**	ACCTGATTTGTTTATATCTGAC
**F114**	TTGTTCGAGGCAGTTGCG
**F115**	TGACAATGGGGTGGAGGG
**F120**	GGGTAGCCTAGACTCATCC
**F121**	ATGCGGAAGTGACTGAGG
**GG1**	CAACAAGAAGACGGAACCTAGGCACCCCAGGCTTTACAC
**GG2**	CAACAAGAAGACGCAAACTTTGCTTTGCCACGGAACGG
**GG3**	CAACAAGAAGACCCGTTTGCGAGGCTAAAGGC
**GG4**	CAACAAGAAGACACGGTTCCCACTCCCAAAGC
**T256**	CGTGCGAGACTCAACCCAAAC
**T257**	GAAACCTGATCGAACCGTTTTAC

Each plasmid for transformation was generated based on two PCR products–one of the plasmid with homologous sequences and one of the *kanR* resistance cassette. For plasmid pFN_7_37_2k_kanRn the kanamycin resistance cassette *kanR* was PCR amplified from pUC4K [36] using the primer pair GG1 / GG2. The plasmid with the long sc_7_37 insert was amplified with the primers GG3 / GG4. The resulting plasmid pFN_7_37_2k_kanRn was generated by the digestion of the PCR products by type IIS restriction enzyme BbsI and subsequent ligation by T4 DNA ligase (both NEB, USA).

Two additional vectors were constructed that contained a version of *kanR* with slightly different 5´ and 3´ UTR. Primer pair F13 / F14 was used for PCR amplification of this *kanR* cassette. This cassette was inserted in the plasmids with the long and the short homologous sc_7_37 sequence, respectively. Both plasmids (with long and short sc_7_37 insert) were amplified with the primer pair F5 / F6. The resulting plasmids are termed pFN_7_37_kanR and pFN_7_37_2k_kanR and were generated by the digestion of the PCR products with XbaI and BmtI and subsequent ligation by T4 DNA ligase (both NEB, USA). Correct insertion of the respective fragments into the pGEM-T backbone was verified by Sanger sequencing. Plasmids for transformation were purified using the midi-prep plasmid purification kit from Macherey Nagel (Düren, Germany).

### Cultivation of *Phormidium lacuna*

*Phormidium lacuna* strains HE10DO and HE10JO were cultivated at 23 °C in f/2 salt water medium [32]—but generally without Na_2_SiO_3_ or in f/2^+^ (in which nitrate and phosphate are 10x increased) under permanent illumination (30 μmol m^-2^ s^-1^ white light from fluorescent tubes Lumilux-DeLuxe L 18/954, Osram, Munich, Germany) and continuous shaking (70 rpm). For agar plates, f/2 medium with 1.5% Bacto Agar (BD Diagnostics, Franklin Lakes, NJ, USA) was used.

### Transformation

For transformation, *Phormidium lacuna* [32] was cultivated in 100 ml f/2 medium to an optical density OD_750 nm_ of 0.25–0.35. The cell suspension was homogenized using an Ultraturrax (Silent Crusher M. Heidolph, Schwabach, Germany) with the dispersion tool 18F at 10,000 rpm for 3 min. The cell suspension was centrifuged at 6000 g and 4°C for 15 min. After each centrifugation step, the supernatant was removed. Cells were resuspended in 20 ml water (4 °C) and centrifuged again. This washing step was repeated. The cells were finally suspended in the residual liquid, transferred into 1.5 ml tubes and centrifuged again at 6000 g at 4°C for 15 min. Cells were finally suspended in 1 ml supernatant. Portions of 100 μl were mixed with 3–30 μg DNA in 10 μl water, transferred into 10 ml f/2 medium, and cultivated for 2 d. Cells were again centrifuged, resuspended in 1 ml medium and transferred to f/2 agar plates with 0, 70, and 120 μg/ml Km. Resistant lines were identified after 10–28 d by light microscopy (single filament and smaller filament bundles) or by eye (bigger filament bundles) through the lid of the closed agar plate. An overview about the protocol is given in Table 2. Resistant cell filaments can be distinguished from wild type cell filaments by color. Relevant regions were marked on the petri dish and the respective cells were transferred to suspension culture under sterile conditions by an inoculating loop. Transgenic cells were cultivated on increasing Km concentrations in f/2^+^ suspension culture until complete segregation of selection marker was achieved. For electroporation experiments, mixtures of cells (as prepared above) and DNA were transferred into 1 mm cuvettes and with 300 V, 2 ms pulses (Gene Pulser Xcell, Bio-Rad, Hercules, CA, USA) before they were transferred into 10 ml f/2 medium and 2 d cultivation step.

**Table 2:** Summary of NT protocol for *Phormidium lacuna*.

	Duration
**Cultivate of 100 ml *Phormidium lacuna* until OD**_**750 nm**_ **= 0.3**	6 d
**Ultraturrax treatment and OD**_**750**_ **measurements**	20 min
**Centrifuge and remove supernatant**	20 min
**Suspend cells in 20 ml H**_**2**_**O and centrifuge, 4° C, repeat 1 x**	40 min
**Concentrate cells by centrifugation, suspend in 1ml (for 10 transformations)**	20 min
**Mix 100 μl cell suspension with 30 μg DNA (from midi prep)**	15 min
**Cultivate in 10 ml f/2**	2 d
**Centrifuge 1 x**	15 min
**Growth on agar plates with and without Km**	2–4 weeks
**Select resistant filaments under microscope, transfer into f/2 with Km**	30 min for each
**Growth in f/2**^**+**^ **with Km**	2 weeks
**PCR test for insertion and segregation**	5 h

### Validation of transformants

To test for homologous integration, ca. 10 mg cell samples (wet weight) were homogenized and lysed mechanically by micropestle and subjected to PCR with Taq Polymerase (NEB, USA). The following primers were used: F25 / F28 for transformants of plasmid pFN_7_37_kanR and F120 / F121 for transformants of either plasmid pFN_7_37_2k_kanR or pFN_7_37_2k_kanRn (see Table 1 for primer sequences). These primers bind only in the *Phormidium lacuna* genome, upstream or downstream of the integration site.

### Bioinformatics

Protein sequences of the NTFs of *Synechocystis* sp. PCC 6803 were obtained from the NCBI data base. NTF sequences of 6 naturally competent cyanobacteria (NCC) were identified by the offline NCBI tool BLAST+ (version: 2.7.1 [37]) based on the annotated NTFs of *Synechocystis* sp. PCC 6803. All NTFs identified in this way were used in a BLAST query against all cyanobacterial sequences of the NCBI non-redundant database (August 2018). Bit score as indicator of homology was processed by a minimum homology quotient method: For each homolog, the bit score of each alignment was divided the by smallest bit score of the respective NCC pairs.

### DAPI fluorescence

Fluorescence was measured with a JASCO FP-8300 fluorimeter (Jasco, Tokio, Japan). Fluorescence emission was usually recorded at 490 nm. For a DNA calibration curve, 100 ng/ml DAPI were dissolved in f/2 growth medium and the fluorescence was recorded. Calf thymus DNA (Sigma-Aldrich, St. Louis, MO, USA) at a given concentration was dissolved and the exact concentration determined by UV/vis absorbance at 260 nm. The DNA was then added at various concentrations to the DAPI solution and the fluorescence recorded again. For the calibration curve, the difference between both spectra at the peak excitation of 363 nm was plotted against the DNA concentration.

*Phormidium lacuna* liquid cultures were homogenized by an Ultraturrax for 3 min at 10,000 rpm. To determine the cell concentration, the total length of all filaments in a given volume of a counting chamber was estimated (about 50 filaments for each sample) and divided by the average cell length (4 μm). Cells were quantitatively disrupted using an Aminco French pressure cell (Thermo Fischer, Waltham, MA, USA). A fluorescence excitation spectrum (300 to 400 nm) was recorded. Thereafter, DAPI was added to a final concentration of 100 ng/ml and the spectrum was measured again. After addition of 5 μl DNase (75 Kunitz) the fluorescence decreased over 30 min—2 h. The difference of the value at the excitation peak of 363 nm before and after DNA digestion was taken as measure for DNA concentration. The DNA concentration *d* in the solution was obtained from the calibration and the multiplication by 41.87/51.30 to correct for the slightly different GC contents of both species (41.87% for bovine and 51.30% for *Phormidium lacuna* sp. HE10JO); GC does not induce DAPI fluorescence [38]. The number *n* of chromosome copies per cell was calculated by

$$n=\frac{d∙a}{m∙g∙c}$$

with Avogadro constant *a* = 6.02 · 10^23^ mol^-1^, molecular mass of one basepair *m* = 660 g (bp mol)^-1^, genome size *g* = 4.8 Mbp, concentration of cells in the measuring solution *c*. A genome size of 4.8 Mb from genome sequencing was taken for *Phormidium lacuna* [32].

## Results

### Transformation of *Phormidium lacuna*

**Fig 1:**
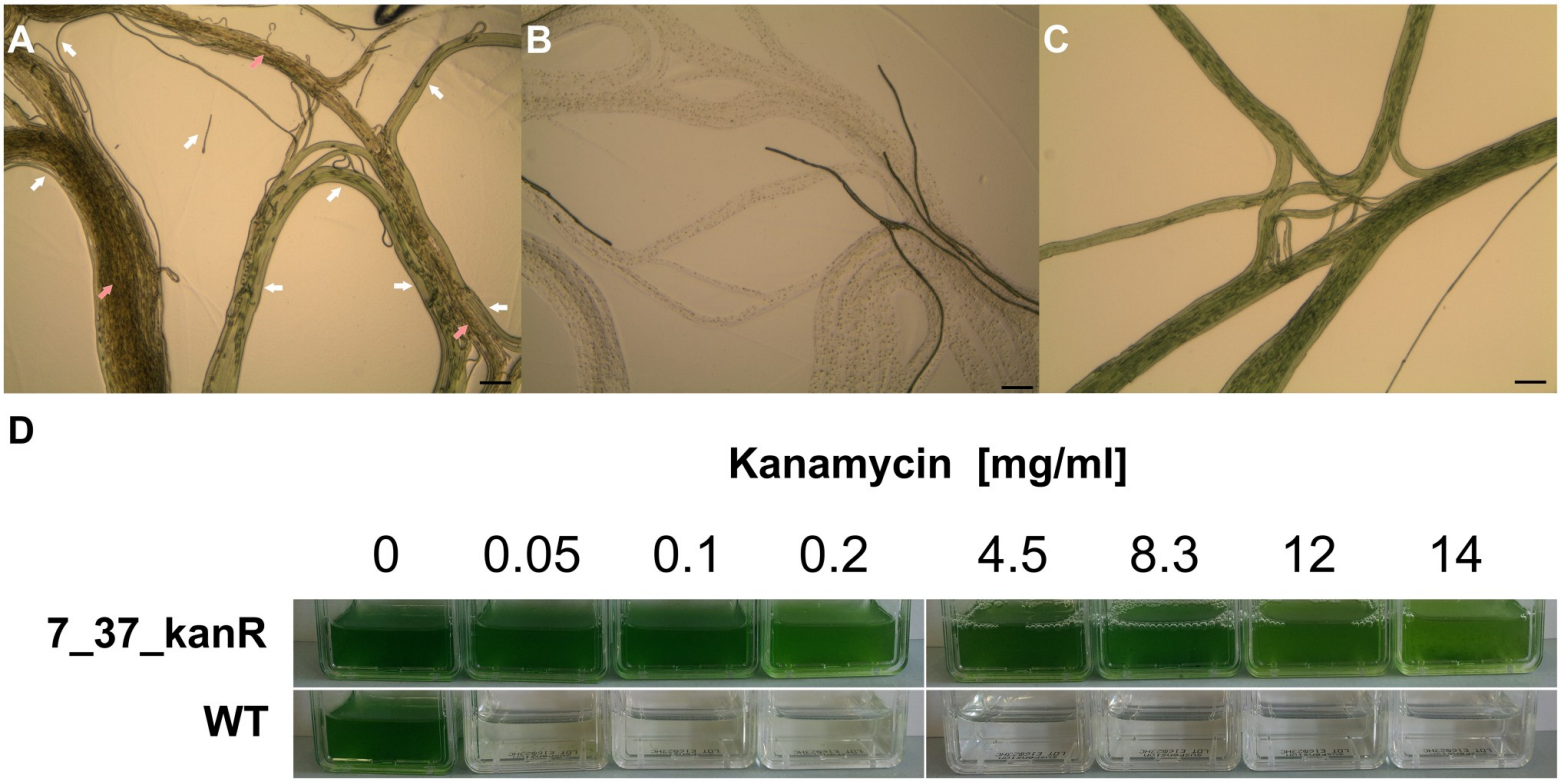
Growth of *Phormidium lacuna* on selection medium. (A-C) Filaments of *Phormidium lacuna* HE10DO after transformation with pFN_7_37_kanR during or after selection on agar plates. (A, B) Two weeks after transformation (A. 70 μg/ml Km; B. 120 μg/ml Km); (C) 4 weeks after transformation, selection on 120 μg/ml Km. In A brownish (red arrows) and greenish (white arrows) filaments can be distinguished, which represent non-transformed and transformed lineages, respectively; in B an area of the agar plate with a high frequency of disintegrated filaments was chosen, the intact filaments are transformed and alive; in C all filaments are alive and transformed. Bundles of parallel filaments are characteristic for growth of *Phormidium lacuna* on agar medium, the growth pattern is comparable to that of wild type on Km-free agar medium. Scale bars 100 μm. (D) Kanamycin resistance of *Phormidium lacuna* HE10JO wild type and pFN_7_37_kanR transformants (liquid cultures 1 week after inoculation). Wild type cannot proliferate at 50 μg/ml Km or above, while pFN_7_37_kanR transformants can grow up to 14 mg/ml Km.

We initially established a transformation protocol for *Phormidium lacuna* strains HE10JO and HE10DO by electroporation that was based on protocols for other filamentous cyanobacteria [39–42]. For integration into cyanobacterial DNA we used homologous recombination, which works efficiently in diverse cyanobacteria such as *Synechocystis* PCC6803, *Synechococcus elongatus* PCC7942, or *Nostoc* Os-1 [43–45]. In the transformation vectors, the homologous sequence “sc_7_37” was interrupted by a *kanR* resistance cassette. sc_7_37 stands for the 37^th^ open reading frame of DNA scaffold 7, the Genbank database entry for the protein is WP_087706519. It is annotated as a hypothetical protein. Since homologous proteins in other cyanobacteria (based on BLAST analysis) are annotated as hydrogenases, we considered this function as relevant in our study. Hydrogenases are oxygen sensitive; oxygenic photosynthesis and hydrogenase activity is usually separated either temporally in non-heterocystous cyanobacteria or spatially in heterocystous species [46]. Since the non-heterocystous *Phormidium lacuna* was cultivated under continuous illumination, we considered an interruption of this open reading frame to have no or minor consequences on growth of the transformants. With the vectors pFN_7_37_kanR, pFN_7_37_2k_kanR, and pFN_7_37_2k_kanRn (see Methods section and below), we obtained Km resistant lines in about 40% of trials in the initial electroporation experiments (Table 3). During these studies we isolated a resistant line from a control experiment with the strain HE10DO in which cells were incubated with DNA, but no electroporation pulse was given. The transformation protocol could be optimized (Table 2 and Methods section) so that in almost all natural transformation assays, DNA was integrated in *Phormidium lacuna* HE10DO cells (Table 3). Fig 1A shows examples for filaments on selection medium at different time points after transformation.

**Table 3:** Transformation of *Phormidium lacuna* HE10DO by electroporation and natural transformation.

Vector	Homologous sequences on either side of *kanR*	Electroporation	Natural transformation
**pFN_7_37_kanR**	500 bp	44% (15 of 34)	67% (2 of 3)
**pFN_7_37_2k_kanR(n)**	1000 bp	44% (8 of 18)	94% (15 of 16)

The numbers stand for successful transformations, i.e. 1 or more resistant lines were isolated in the relevant trial. pFN_7_37_2k_kanR(n) refers to two plasmids with longer homologous sequences—pFN_7_37_2k_kanR and pFN_7_37_2k_kanRn.

**Fig 2:**
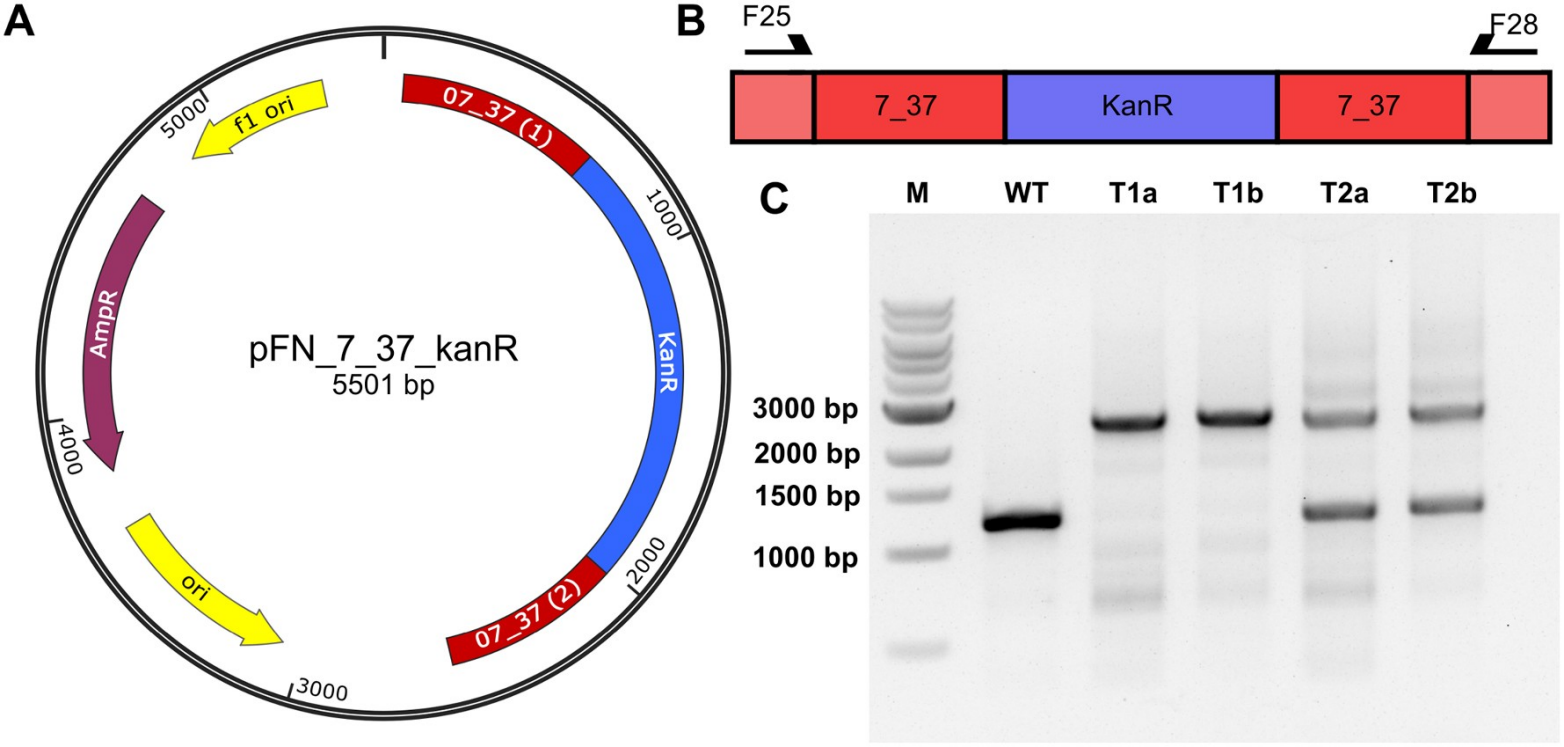
Validation of *Phormidium lacuna* HE10DO transformants by PCR. (A) Plasmid map of pFN_7_37_kanR, red—homologous sequences of the sc_7_37 locus, blue—kanamycin resistance cassette *kanR*, yellow—origin of replication (f1—bacteriophage origin, other—pUC origin for *E*. *coli*), purple—ampicillin resistance cassette; (B) Integration site of pFN_7_37_kanR and primer binding sites. red—homologous sequences encoded on the vector, pale red—*Phormidium lacuna* chromosome, blue—kanamycin resistance gene. Primer pair: F25/F28 covering whole insertion site; (C) agarose gel for the PCR with the primer pair that covers the full insert for *Phormidium lacuna* HE10DO WT and pFN_7_37_kanR transformants. Transformants T1a and T1b were cultivated after selection on agar plate for one cultivation period (7 days) in f/2^+^ liquid medium with 250 μg/ml Km and one period in f/2^+^ with 1000 μg/ml Km. T2a and T2b were cultivated only for one period at 250 μg/ml Km. M: 1kb DNA ladder (NEB, USA). Integration of the *kanR* cassette into the genome of *Phormidium lacuna* is indicated by the larger PCR product (2560 bp), the native sequence in indicated by the small PCR product (1213 bp).

Plasmid pFN_7_37_kanR has short ca. 500 bp homologous sequences flanking the *kanR* resistance cassette on each side, whereas pFN_7_37_2k_kanR and pFN_7_37_2k_kanRn have ca. 1000 bp homologous sequences on each side. The two bigger plasmids differed only in the 5’ and 3’ UTR of the selection marker and were therefore summarized as pFN_7_37_2k_kanR(n). A scheme of pFN_7_37_kanR is shown in Fig 2A. In a comparison of electroporation and NT that were performed under similar conditions, the success rate was always higher for NT as compared to electroporation (Table 3). When the pFN_7_37_2k_kanR(n) vectors were used, 15 out of 16 NT transformation trials were successful, i.e. resulted in the isolation of resistant lines that could be confirmed by PCR (see below). In the electroporation experiments, only 44% transformation trials were successful. We assume that the deleterious effect of the electric pulse overrides the positive effect that the pulse might have on DNA incorporation into the cells. We can even not rule out that in the electroporation experiments that DNA is taken up by NT only and that the electropulse has only deleterious effects. For transformation of *Synechocystis* sp. PCC 6803 it was observed that longer flanking sequences are beneficial for homologous integration into the genome [47]. For *Phormidium lacuna*, we found no significant difference of transformation success between 500 bp and 1000 bp flanking sequences: rates were the same in the electroporation trials and the number of replicates in this comparison were too low in the NT experiments (Table 3). Since longer homologous sequences had at least no negative effect on transformation in electroporation experiments, we performed most of the NT experiments only with the bigger plasmids. The experiments presented in Table 3 were performed with *Phormidium lacuna* strain HE10DO. Additional electroporation experiments, which resulted in resistant transformants, were also performed with strain HE10JO.

### Strong resistance against kanamycin

We found that transformants of *Phormidium lacuna* HE10JO and HE10DO with the *kanR* cassette were resistant against very high Km concentrations. Transformed lines could be routinely cultivated up to 14 mg/ml Km (Fig 1B and S1 Fig). *Escherichia coli* DH5α and *Synechocystis* sp. PCC 6803 cells, transformed with the same *kanR*, were resistant up to ca. 200 μg/ml and ca. 500 μg/ml Km, respectively. The upper limit of the Km resistance of transformed lines was repeatedly detected between 14 mg/ml and 30 mg/ml Km; at 30 and 50 mg/ml Km *Phormidium lacuna* transformants could not proliferate anymore (S1 Fig). In an extensive literature survey we found a report about an environmental *Enterococcus* strain that was resistant up to 2 mg/ml Km [48], but no report about higher Km resistance. Thus, transformed *Phormidium lacuna* has probably the strongest Km resistance reported so far. Besides the Km resistance the *Phormidium lacuna* transformants showed no apparent phenotype.

### Selection of homozygous mutants

The integration into the genome was validated by PCR. Fig 2 shows results from a transformation with pFN_7_37_KanR. The used primers bind to regions in the genome of *Phormidium lacuna* just upstream or downstream of the insertion site, respectively (Fig 2B). In wild type extracts, an expected short PCR product of 1213 bp was detected (Fig 2C). Lines T1a and T1b are from filaments transformed with pFN_7_37_KanR that had been cultivated in liquid medium with 250 μg/ml and 1000 μg/ml kanamycin after transformation and the expected long PCR product with 2560 bp was detected. In T2a and T2b, which are from filaments transformed with pFN_7_37_KanR that were cultivated for 9 d in liquid culture with 250 μg/ml Km, both the 1213 bp and the 2560 bp PCR products were present. The integration of *kanR* into the chromosome of *Phormidium lacuna* transformants was confirmed by PCR for all selected transformants. When the PCR tests were performed after prolonged cultivation on high Km concentration (like 1000 μg/ml), the pattern of T1a/T1b was obtained (Fig 2C). In 8 independent transformation experiments the pattern of T2a/T2b was obtained, which indicates the presence of the wild type chromosome and the recombinant chromosome with the *kanR* insertion at the same time. A possible explanation for this pattern is that *Phormidium lacuna* has more than one chromosome copy, i.e. that it is polyploid as reported also for several other cyanobacteria [25]. *kanR* could be first integrated in a subfraction of the chromosomes after transformation. Prolonged selection on antibiotics results in an increase of the fraction of targeted chromosomes until finally all chromosomes bear the insertion and homozygous transformants are selected. Tests for the presence of the vector backbone using PCR primers for the ampicillin resistance cassette showed negative results.

### Ploidy of *Phormidium lacuna*

**Fig 3:**
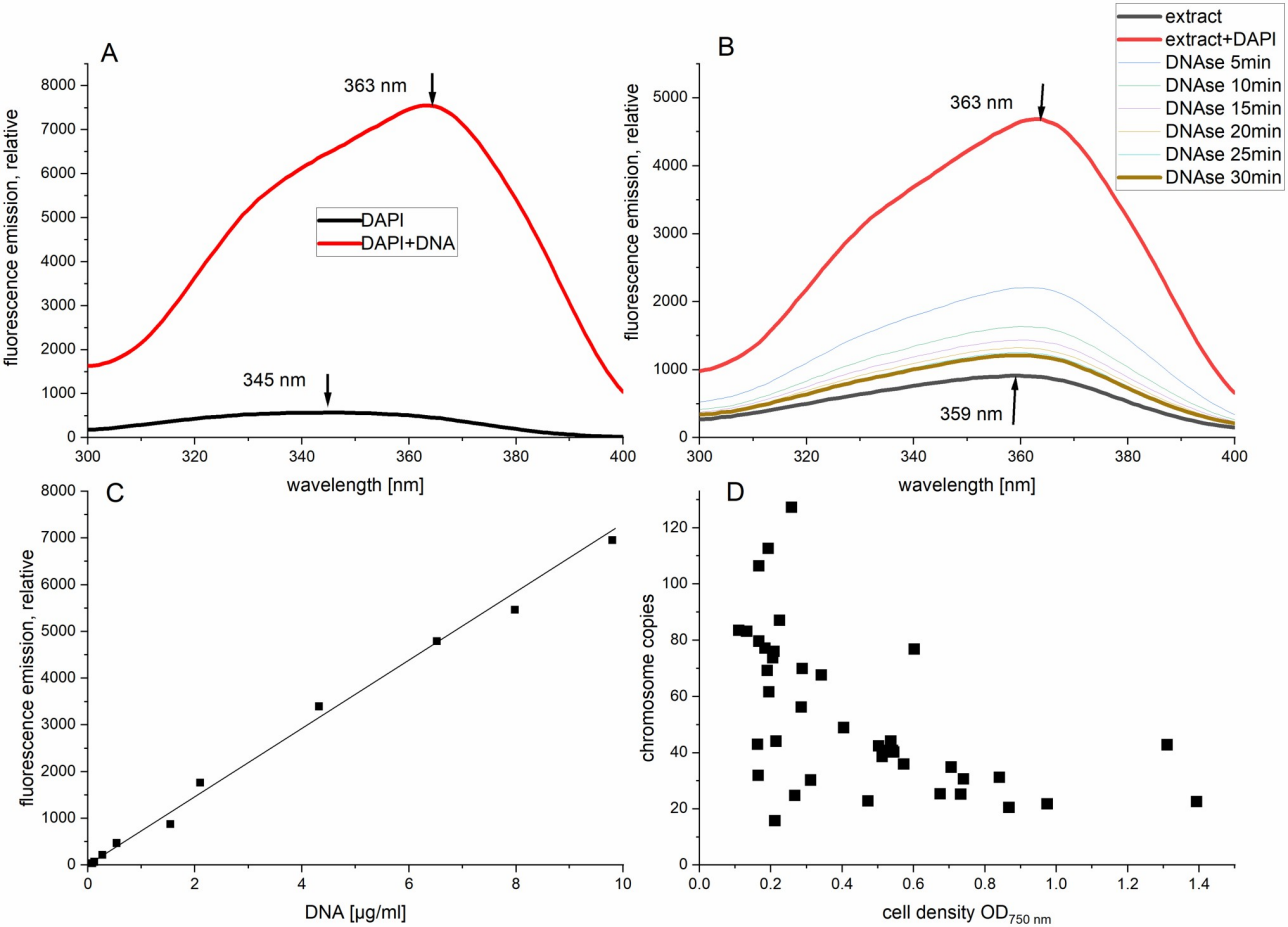
DAPI assay to determine DNA concentrations in *Phormidium lacuna* HE10DO extracts. (A, B) excitation spectra from 300 nm to 400 nm, fluorescence emission were measured at 470 nm. (A) spectra of DAPI (black) and DAPI with calf thymus DNA (red); excitation maxima are indicated by arrows; (B) spectra of *Phormidium lacuna* extract (thick line, black), extract after addition of DAPI (thick line, red) and after DNAse addition, measured at intervals as given in the legend (thin lines, various colors), after 30 min, fluorescence was constant (thick line, brown), excitation maxima of extract and extract+DAPI are indicated; (C) calibration curve, fluorescence of DAPI with calf thymus DNA as used for calculation of DNA concentrations, correlation coefficient 0.998; (D) number of chromosome copies of *Phormidium lacuna* calculated according to the methods section and plotted against cell densities, correlation coefficient and slope of a linear regression are 0.3 and -41, respectively, the number of cells is 22 Mio / OD_750 nm_.

In order to find out whether *Phormidium lacuna* cells are also polyploid, we estimated the number of chromosome copies by a DAPI based fluorescence assay. For these measurements, we used extracts of *Phormidium lacuna* HE10DO wild type cells that were cultivated in f/2 medium for 2 to 7 days. DAPI is used for DNA detection in microscopy [49] and extracts [50]. The staining is based on intercalation into double stranded DNA that results in a strong increase of fluorescence quantum yield. Double stranded RNA and DAPI are also fluorescent [51]. RNA did only slightly interfere with our assay. In preliminary experiments we found that the fluorescence of DAPI added to a *Phormidium lacuna* extract (details as described in the Methods section) is only slightly reduced by RNase addition, these changes were in the range of 10%. DAPI (without DNA) had an excitation maximum at 345 nm in our assay, the addition of calf thymus DNA resulted in a major increase of fluorescence with an excitation at 363 nm (Fig 3A). The background fluorescence of the *Phormidium lacuna* cell extract without DAPI was monitored in each set of measurements. The excitation maximum of the extract was 359 nm (Fig 3B). Fluorescence was drastically increased upon addition of DAPI and the excitation maximum was shifted to 363 nm. This maximum wavelength is identical with that of pure DNA and DAPI (Fig 3A). This shows again that RNA does not contribute in a significant extent to the fluorescence spectrum of extract and DAPI, since the DNA and RNA DAPI fluorescence spectra have different maxima [51]. To further distinguish a DNA-specific signal from background fluorescence and from the signal of free DAPI, we included a DNase treatment in the assay. This resulted in a loss of most of the fluorescence signal in each sample (Fig 3B). We used the difference before and after complete DNase digestion as measure for the DNA concentration, for the calculation of DNA concentration we used calibration measurements with calf thymus DNA (Fig 3C). Details for calculation of DNA concentration and chromosome numbers per cell are given in the methods section.

The calculated number of chromosomes per cell differs and is for most measurements between 20 and 90 (Fig 3D). Although for some values of OD_750 nm_ (which indicates cell density and culture age as well) different chromosome copies are measured, especially at low cell density, it is apparent that the copy number reduces at higher OD_750 nm_. Despite the variations we assume that our method is precise enough to say that *Phormidium lacuna* is polyploid (chromosome copy numbers between 20 and 90) and that it can vary due to culture conditions, as demonstrated for OD_750 nm_. Strong variation of chromosome copy number was reported for *Synechocystis* sp. PCC 6803 in dependency on growth phase and environmental factors [29] and a chromosome copy number of more than 600 was reported for the filamentous *Trichodesmium* e*rythraeum* IMS 101 [52].

### Selection of homozygous transformants

**Fig 4:**
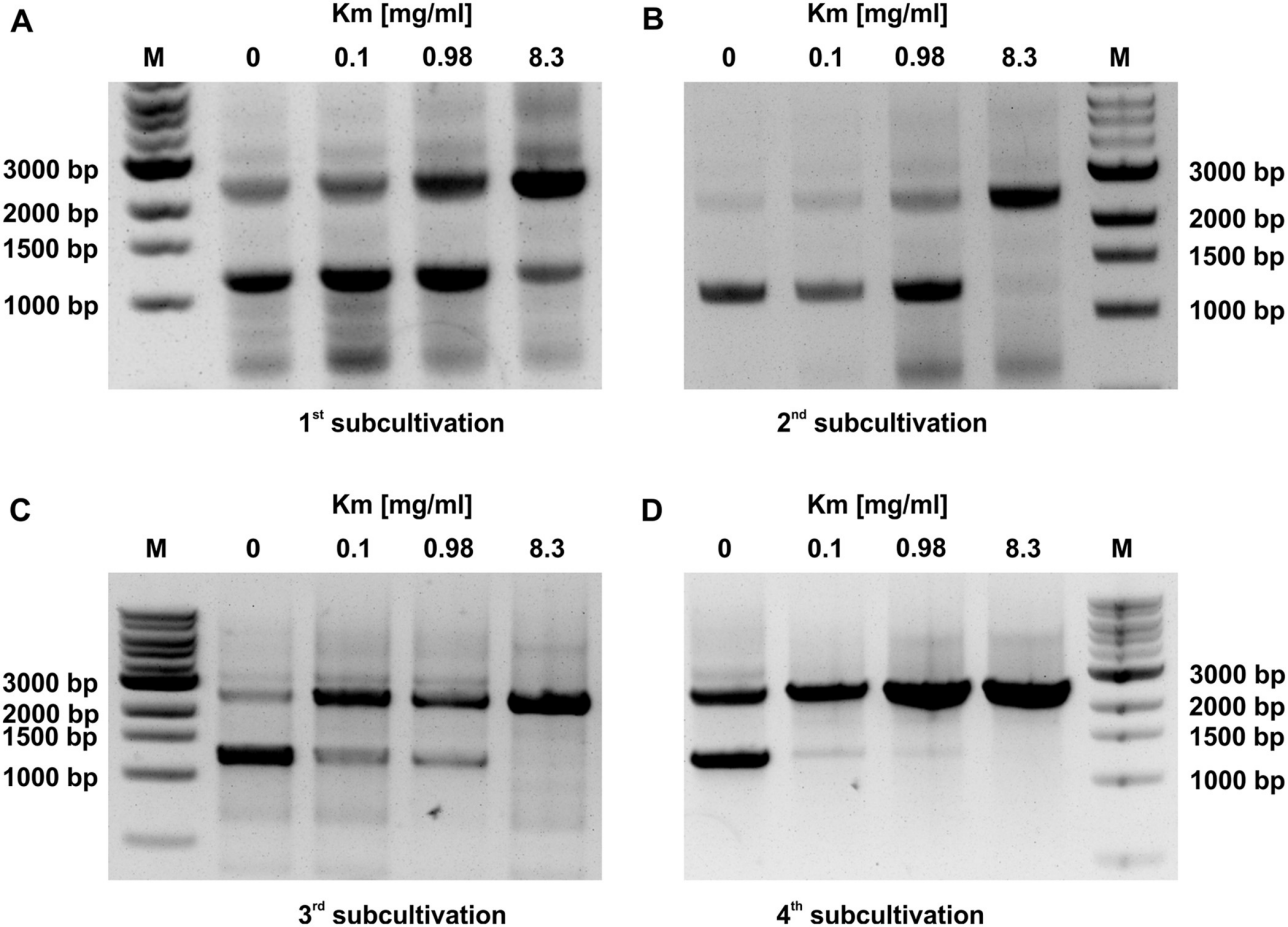
Detection of wild type and recombinant chromosomes in kanamycin resistant *Phormidium lacuna* HE10DO pFN_7_37_kanR transformants with PCR. (A-D) Following cultivation in 0.1 mg/ml Km until 2 weeks after transformation, the sample was divided and subcultivated one to four times on different Km concentrations (A, 0 mg/ml; B, 0.1 mg/ml, C, 0.98 mg/ml; D, 8.3 mg/ml). Primers: F25, F28. PCR product length: native– 1213 bp, recombinant– 2560 bp. Marker: 1 kb DNA ladder (NEB, USA).

We also studied how the Km concentration in the medium affects the selection of homozygous transformants by detection of *kanR* in the genome via PCR (Fig 4). After transformation of HE10DO with pFN_7_37_KanR, selection of a resistant line, and one cultivation cycle in suspension culture with 100 μg/ml Km, the filaments were divided and cultured at 0, 0.1, 0.98 and 8.3 mg/ml Km in suspension culture with subcultivation every 7 d for 4 weeks. After the first subcultivation, two PCR bands were observed in all cultures, indicating that Km resistance was integrated in a part of the chromosomes but not in all (Fig 4A). Without antibiotic pressure, the 2560 bp PCR product decreased transiently and increased again after the 4^th^ subcultivation (Fig 4B–4D), and the 1213 bp wild type PCR product was present through all subcultivations. In the 100 μg/ml Km samples, the wild type band was diminished after the third subcultivation and almost, but not completely, lost after the 4^th^ subcultivation. The results were similar for the selection on 980 μg/ml Km. With 8300 μg/ml Km, the wild type band disappeared almost completely already after the 2^nd^ subcultivation and was apparently lost after the 3^rd^ and 4^th^ subcultivations. Thus, high concentrations of Km result in rapid segregation of *kanR* into all chromosomes. The high Km resistance of *Phormidium lacuna* transformants could provide an advantage for fast selection of homozygous mutants.

### Prediction of potentially naturally competent cyanobacteria

*Phormidium lacuna* HE10DO is the first filamentous non-heterocystous cyanobacterium for which a NT protocol was established. According to mutant studies with *Synechocystis* sp. PCC 6803, proteins of the type IV pili PilA1, PilB1, PilD, PilM, PilN, PilO, PilQ, PilT1 and the DNA receptor ComEA and ComF are NTFs, i.e. required for NT [19–22]. Other NTFs that are essential for NT in general (but for this was not explicitly demonstrated by knock-out mutants) are ComEC, DprA, and RecA [18]. A functional type IV pilus in combination with this set of expressed non-pili proteins is the essential prerequisite for NT. We can therefore assume that the probability for NT is high if a species has functional homologs of all proteins. The protein sequences of *Synechocystis* sp. PCC 6803, which were proven or predicted to be essential for natural transformation, were received from the NCBI database. In order to get an overview about the distribution of NTF in selected cyanobacteria, first the selected protein sequences of *Synechocystis* sp. PCC 6803 were used as query to identify the NTF with BLASTp in the other naturally competent cyanobacteria (NCC) *Synechococcus elongatus* PCC 7942, *Synechococcus* sp. PCC 7002, *Thermosynechococcus elongatus* BP-1, *Microcystis aeruginosa* PCC 7806, and *Phormidium lacuna* HE10JO [7–12]. The strains *Phormidium lacuna* HE10JO and HE10DO are very similar and the genomic data for HE10JO is considered also relevant for the strain HE10DO [32], see also Methods section. This set of proteins of all NCC is used to define a range of similarity to decide whether other homologs could be functional for NT or not. We compared all NTFs of all NCCs with the predicted proteins of the genomes of selected other cyanobacteria using BLASTp. In order to normalize each target, the highest bit-score (out of 6) was divided by the lowest bit-score among the 6 NCCs (i.e. the most unrelated NTF pair among NCCs). If the quotient is ≥ 1, we regard the target protein as functional homolog, because it is then within the range of NCC. More precisely, a value above 1.0 indicates that are protein shares a higher homology with at least one of the NCC than this protein shares among the most distantly related species inside the NCC group. If all NTF homologs of a species have values ≥ 1, the chances are high that all NTFs are functionally present in this species. Among 28 cyanobacterial species for which no NT is reported, 19 have quotients ≥ 1 for all 13 NTF homologs and are thus promising candidates for NT (Table 4). Among them are 14 filamentous cyanobacteria including two members of the genera *Arthrospira / Spirulina* and *Trichodesmium* with high economic or ecological impact. For other species the quotient for one or more NTFs is below 1, yet all essential genes for NT seem to be present in the genome but in lower homology to the NCC. These organisms may also be naturally transformable but lower likelihood of success. Quotients ≤ 0.2 are considered as indicative for random hits during the BLAST search. Therefore, the 3 species that have at least one quotient ≤ 0.2 are regarded as critical for NT.

**Table 4:** Prediction of cyanobacteria that are potentially naturally competent.

NTFs	ComEA	ComEC	ComF	DprA	PilA1	PilB1	PilD	PilM	PilN	PilO	PilQ	PilT	RecA	PC/NCC
Strains														
***Gloeobacter violaceus* PCC 7421**	0.9	1.1	1.2	0.9	1.3	0.7	0.8	1.0	1.1	0.7	1.4	1.0	0.9	
***Leptolyngbya boryana* PCC 6306**	1.4	1.6	1.7	1.2	1.3	1.1	1.4	1.5	1.8	1.5	2.6	1.3	1.0	PC
***Synechocystis sp*. *PCC* 6803**	2.9	5.2	3.8	2.8	5.6	1.6	2.4	2.3	7.2	6.2	8.8	1.4	1.4	NCC
***Synechocystis* sp. PCC 7509**	1.1	1.8	1.8	1.3	1.0	1.1	1.5	1.6	1.5	2.0	2.7	1.3	1.0	PC
*Prochlorococcus marinus* (*)	0.1	0.2	0.3	0.1	0.5	0.0	0.2	0.1	0.5	0.4	0.2	0.1	1.1	
***Pseudanabaena* sp. PCC 7367**	1.1	1.6	1.4	1.0	1.8	0.9	1.3	1.3	1.2	0.9	1.9	1.1	1.1	
***Cyanobium gracile* PCC 6307**	0.9	0.6	0.4	0.4	1.0	0.7	0.8	0.1	0.9	0.4	1.3	0.5	1.1	
***Synechococcus elongatus* PCC 7942**	2.5	7.0	5.0	2.6	5.8	1.7	2.5	2.6	7.5	6.8	6.9	1.5	1.3	NCC
***Synechococcus* sp. PCC 7002**	2.7	7.3	3.8	2.6	7.1	1.6	2.6	2.4	6.4	8.7	8.9	1.3	1.3	NCC
***Synechococcus* sp. PCC 7502**	1.1	1.6	1.6	1.3	1.3	0.9	1.2	1.3	1.5	1.2	2.1	0.5	1.0	
***Thermosynechococcus elongatus* BP-1**	2.8	5.9	5.0	2.5	3.8	1.7	2.4	2.7	7.8	6.8	8.9	1.4	1.3	NCC
***Geitlerinema* sp. PCC 9228**	1.4	2.0	1.8	1.3	1.4	1.1	1.4	1.6	1.8	1.1	3.1	1.2	1.1	PC
***Cyanothece* sp. ATCC 51142**	1.5	1.7	2.1	1.5	2.4	1.3	1.5	1.9	2.4	2.5	3.1	1.2	1.2	PC
***Arthrospira platensis* NIES-39**	1.4	1.7	1.9	1.4	1.2	1.1	1.4	1.6	1.9	1.7	2.6	1.3	1.1	PC
***Trichodesmium erythraeum* IMS101**	1.4	1.6	1.6	1.4	1.6	1.0	1.5	1.5	1.6	1.4	2.5	1.1	1.1	PC
***Oscillatoria acuminata* PCC 6304**	1.4	1.7	1.9	1.3	1.8	1.1	1.4	1.6	1.7	1.1	2.8	1.2	1.1	PC
***Oscillatoria nigro-viridis* PCC 7112**	1.4	1.6	2.0	1.4	1.4	1.1	1.3	1.6	1.8	1.2	2.6	1.2	1.1	PC
***Phormidium ambiguum* IAM M-71**	1.4	1.7	1.7	1.4	0.9	1.1	1.2	1.6	1.9	1.8	1.9	1.2	1.1	
***Phormidium lacuna* HE10JO**	2.5	5.8	4.0	2.5	4.0	1.7	2.6	2.6	6.2	8.1	7.7	1.5	1.4	NCC
***Phormidium* sp. OSCR**	2.3	5.0	3.3	2.1	3.2	1.6	2.4	2.6	5.8	7.1	6.0	1.5	1.4	PC
***Phormidium tenue* NIES-30**	1.1	1.4	1.5	1.2	1.2	1.1	1.3	1.6	1.6	1.2	2.7	1.2	1.1	PC
***Phormidium willei* BDU 130791**	2.4	5.6	3.7	2.5	3.9	1.6	2.5	2.6	6.1	8.0	7.5	1.5	1.4	PC
***Geminocystis herdmanii* PCC 6308**	1.1	1.6	1.9	1.4	2.1	1.1	1.4	1.5	2.4	1.7	1.7	1.2	1.1	PC
***Microcystis aeruginosa* NIES-843**	2.4	3.9	3.1	2.4	2.8	1.5	2.1	1.9	3.8	4.8	5.4	1.3	1.3	PC
***Microcystis aeruginosa* PCC 7806**	2.6	4.3	3.2	2.5	5.6	1.6	2.4	2.5	5.2	6.4	6.4	1.4	1.4	NCC
***Stanieria cyanosphaera* PCC 7437**	1.2	1.8	1.8	1.4	2.3	1.2	1.4	1.5	1.8	2.1	3.4	1.2	1.1	PC
***Spirulina major* PCC 6313**	1.2	1.5	1.7	1.3	1.1	1.1	1.2	1.8	1.4	1.4	2.0	1.1	1.1	PC
***Chroococcidiopsis thermalis* PCC 7203**	0.9	1.7	1.7	1.4	1.6	1.2	1.5	1.4	1.8	2.0	2.8	1.3	1.0	
***Fischerella muscicola* PCC 7414**	0.1	1.8	1.9	1.4	1.8	1.1	1.3	1.6	2.1	1.8	2.9	1.3	1.1	
***Anabaena cylindrica* PCC 7122**	1.4	1.8	1.8	1.4	1.3	1.1	1.4	1.6	1.8	1.7	3.0	1.3	1.1	PC
***Anabaena* sp. 90**	1.4	1.8	1.9	1.4	1.3	1.1	1.3	1.5	2.0	1.9	2.3	1.2	1.0	PC
***Nostoc punctiforme* PCC 73102**	0.9	1.8	1.8	1.4	1.3	1.1	1.4	1.6	1.9	1.8	2.7	1.3	1.1	
***Nostoc* sp. PCC 7120**	1.4	1.8	1.8	1.4	1.7	1.1	1.3	1.5	2.0	1.9	2.9	1.3	1.1	PC
***Calothrix* sp. PCC 6303**	1.3	1.8	1.7	1.3	1.6	1.1	1.3	1.7	1.8	1.5	2.9	1.3	1.0	PC

The minimum bit score quotient of (putative) natural transformation factors (NTFs) of 34 cyanobacterial species are summarized. In the first step, NTF homologs of 6 naturally competent cyanobacteria (indicated by NCC in the last line) were identified. These NTFs were used as queries in a BLAST search against protein sequences of 28 cyanobacterial species. The bit score of each homology hit was divided the by smallest bit score of the respective NCC pairs resulting in the minimum bit score quotient. Therefore a quotient above 1.0 indicates that a protein sequence shares a higher homology with at least one of the NCC than the most distantly related species among the NCC group share with each other. If all values are 1.0 or higher the respective organism is predicted as potentially naturally competent (PC). For some NTFs the homology between the respective species and the NCC is lower (indicated by a quotient between 0.2 and 1.0), yet BLAST results seem to be specific and the respective NTF seem to be present. These organisms may also be naturally transformable but lower likelihood of success. Quotients ≤ 0.2 are considered as indicative for random hits during the BLAST and the respective species are predicted to be not naturally competent. (* full strain name: *Prochlorococcus marinus* subsp. marinus str. CCMP1375).

## Discussion

Even though NC is known for several single celled cyanobacterial species and one filamentous cyanobacterium, *Nostoc muscorum* [15], this mechanism was considered as rare trait among cyanobacteria [6, 13, 14]. The finding of NC in *Phormidium lacuna*, the first filamentous non-heterocystous cyanobacterium, was therefore unexpected and surprising. Based on this finding we assume that NC is more common among cyanobacteria. Our bioinformatic studies on the distribution of NTF homologs among 29 species, which are intended to be a representative selection of the cyanobacterial phylum with a focus on filamentous non-heterocystous cyanobacteria, supports this hypothesis: functional homologs of all NTFs are present in at least 19 species. Thus, NC seems to be a widely distributed physiological function in cyanobacteria and could contribute more to evolutionary adaptations than previously suggested. It might play a major role in horizontal gene transfer, genetic recombination, and DNA repair in this phylum. Natural transformation is easy and uncomplicated and a more widespread use would be a benefit for basic and applied research in the field.

Our bioinformatic analysis gives example that the rapidly increasing numbers of cyanobacterial genomes will help to identify potentially transformable species and offers a simple way to rank potential candidates for their probability of NT based on the sequence homology to strains already described as natural competent. Two recently published works [24] also demonstrate that genes for NC are frequent in the cyanobacterial phylum. We chose a different selection of proteins for our analysis. We did not considered proteins, which were demonstrated to be involved in NC in certain cyanobacteria species but whose function in NC is either not completely understood, or which play a role in NC regulation that might be species specific (see also below). We focused on proteins, whose function is understood for NC and which seem to be universal in gram negative bacteria to test for the essential prerequisite for NC. This includes the type IV pilus and competence proteins but also additionally the proteins RecA and DprA, which are not directly included in the DNA uptake into the cell but are essential for the subsequent recombination [18]. RecA is found is all analyzed strain, which is not surprising since RecA-like proteins are nearly ubiquitous in bacteria [53], and are also present in archaea and eukaryotes [54]. DprA is present in all species in our analysis except the *Prochlorococcus* strain, which misses also most other NTFs. DprA is highly conserved in naturally competent species [55] and is thus an essential factor to predict NC together with the competence and pilus proteins. We want to point out that the presented analysis can be used as first indication if a certain species might be naturally competent or not but it is no definitive prediction. If a particular interest exists for a species that is predicted as non competent, one could consider a fresh and/or deeper analysis (for example under consideration of updates or reformation of the respective databases). On the other side not only the complete set of essential genes is relevant for NC but also their coordinated expression is important. Thus, even if the complete set for NC is present in an organism, the physiological state of NC may be hard to stimulate. Gene expression of NTFs is probably dependent on internal and environmental parameters. Molecular studies on the regulation of NC are concentrated on the gram negative *Vibrio cholerae* and the gram positive genera *Bacillus* and *Streptococcus* [55, 56]. For the cyanobacterium *Synechococcus elongatus* PCC 7942 it was demonstrated recently that the circadian clock controls the expression of NTFs [23]. While in *Synechococcus elongatus* PCC 7942 transformation efficiency peaks at dusk and early dark phase, transformation efficiency in *Synechocystis* sp. PCC 6803 is higher when cells are incubated under light instead of darkness [47]. This illustrates that the uptake machinery for DNA via NC might be conserved in the cyanobacterial phylum, but the regulation of NC might be more diverse.

*Phormidium lacuna* filaments are highly motile on agar medium [32] and motility of cyanobacteria is thought to be dependent on type IV pili [57], the same structure that mediates NT. Motility indicates that *pil* genes are expressed, and a preselection of motile strains or conditions that increase motility could help in successful NT. The filamentous growth and motility on agar surfaces and different membranes is the reason why no colonies are obtained after transformation and the respective frequencies cannot be calculated as cfu/μg of plasmid DNA.

Several negative factors could reduce or abolish DNA transfer, such as extracellular polymeric materials, different types of nucleases, and CRISPR/Cas systems. However, DNA is taken up in single stranded form and bound by single strand binding proteins, DprA, and RecA [18] before its integration into the genome. Intracellular DNA during NT could be thus less vulnerable to negative factors that target double stranded DNA such as the restriction endonucleases. Gene transfer into *Nostoc* sp. PCC 7120 by conjugation or into *Arthrospira platensis* C1 by electroporation was improved if DNA was protected from restriction endonucleases by methylation or by the use of suitable inhibitors [58, 59]. Genes for single strand exonuclease RecJ [60], for several endonucleases, and for CRISPR/CAS [61–63] are present in the genome of *Phormidium lacuna* and widely distributed among other cyanobacteria including also other naturally competent species.

In principle, transformation success can be improved by stimulating positive factors or by inhibiting negative factors. In the present *Phormidium lacuna* transformation protocol the cell suspension is homogenized and washed subsequently, this treatment could reduce extracellular transformation barriers as extracellular polymeric materials or extracellular nucleases [5] and thereby promote NT. In NT experiments with other species, the use of more DNA, additional DNA methylation, or temperature variations could inhibit other negative factors.

## Conclusions

We have shown that a newly isolated cyanobacterium *Phormidium lacuna* can be transformed by natural transformation during which a *kanR* resistance cassette is integrated into the genome by homologous recombination, that the transformants are extraordinarily resistant against Km, and that this cyanobacterium has multiple chromosome copies. The established protocol includes few washing steps, DNA addition and subcultivation, and homozygous transformants are obtained after about 4 weeks. Genetic engineering is now possible for another cyanobacterium, which can be used in the future for basic research and biotechnological applications. We hope that our results stimulate trials on natural transformation of other cyanobacteria and thereby contribute to a broadening of research on more species, especially the filamentous ones.

## Supporting information

S1 Fig Kanamycin limit for *Phormidium lacuna* HE10JO pFN_7_37_kanR transformants. Cultures were inoculated to OD750 = 0.2 and were cultivated for 1 week at standard growth conditions. Km concentrations are given above and below. (A) Liquid cultures of one example after 1 week inoculation at given Km concentration. *Phormidium lacuna* HE10DO wild type (WT) and pFN_7_37_kanR transformants. (B) Quantification of OD_750 nm_ as measure cell density. ΔOD750 nm was calculated by the difference of the between the values at day 7 and the start OD_750 nm_. Negative ΔOD_750 nm_ indicates that cells died during inoculation. Mean values +- SE, n = 3. T-test error probabilities p for transformation efficiency are indicated by *, p < 5% and **, p < 0.5%.
(DOCX)

S1 Original Images (ZIP)
