# Supplementary material for: Natural transformation of the filamentous cyanobacterium Phormidium lacuna
Source: PLoS One. 2020 Jun 12;15(6):e0234440. doi: 10.1371/journal.pone.0234440 (PMC7292380; doi:10.1371/journal.pone.0234440)
Supplement: S1 Fig — Cultures were inoculated to OD750 = 0.2 and were cultivated for 1 week at standard growth conditions. Km concentrations are given above and below. (A) Liquid cultures of one example after 1 week inoculation at given Km concentration. Phormidium lacuna HE10DO wild type (WT) and pFN_7_37_kanR transformants. (B) Quantification of OD750 nm as measure cell density. ΔOD750 nm was calculated by the difference of the between the values at day 7 and the start OD750 nm. Negative ΔOD750 nm indicates that cells died during inoculation. Mean values +- SE, n = 3. T-test error probabilities p for transformation efficiency are indicated by *, p < 5% and **, p < 0.5%. (DOCX) [file pone.0234440.s001.docx]

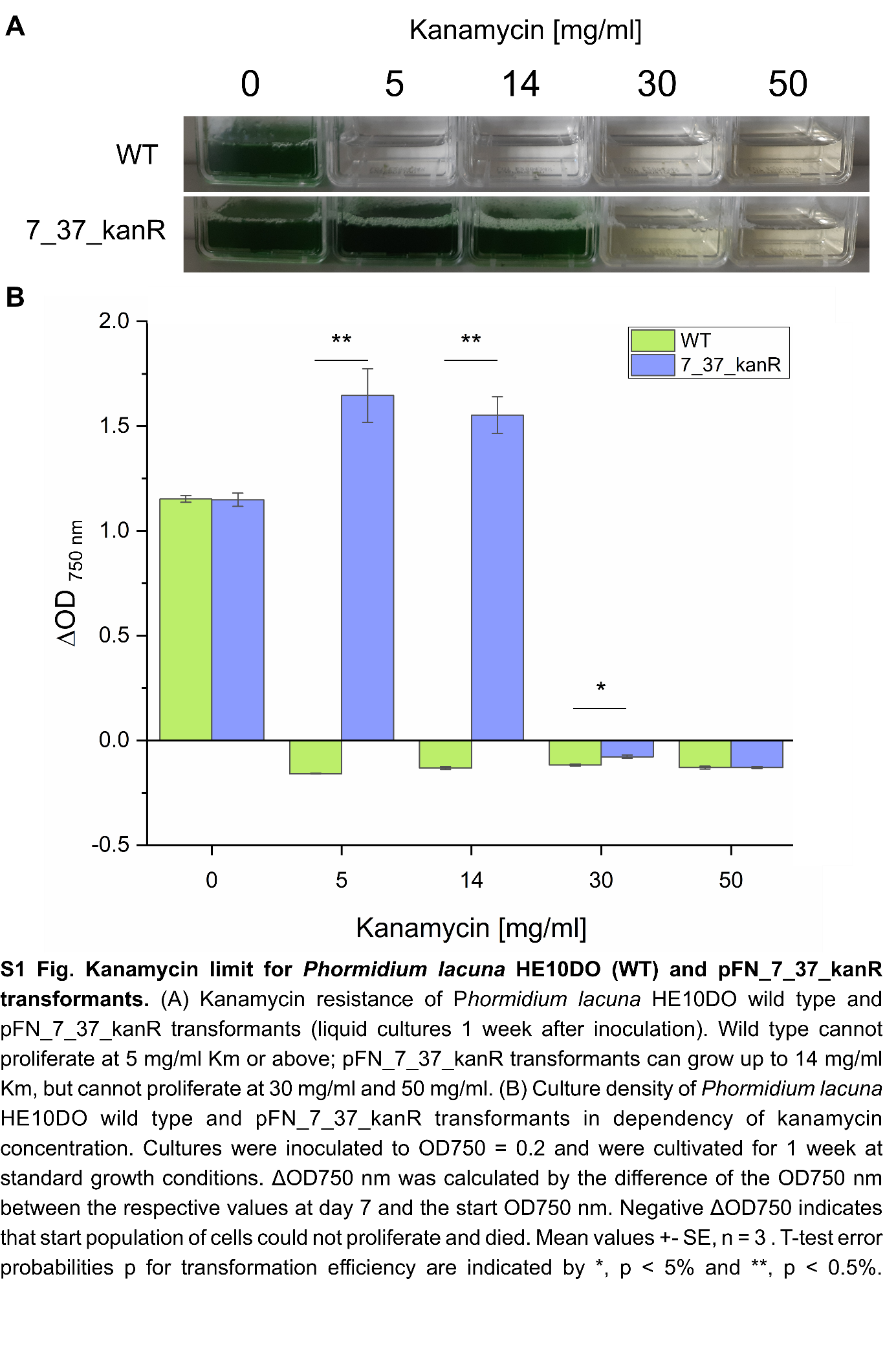


**S1 Fig. Kanamycin limit for *Phormidium lacuna* HE10DO (WT) and pFN_7_37_kanR (7_37_kanR) transformants.** Cultures were inoculated to OD750 = 0.2 and were cultivated for 1 week at standard growth conditions. Km concentrations are given above and below.

(A) Liquid cultures of one example after 1 week inoculation at given Km concentration. *Phormidium lacuna* HE10DO wild type (WT) and pFN_7_37_kanR transformants. (B) Quantification of OD_750 nm_ as measure cell density. ΔOD750 nm was calculated by the difference of the between the values at day 7 and the start OD_750 nm_. Negative ΔOD_750 nm_ indicates that cells died during inoculation. Mean values +- SE, n = 3 . T-test error probabilities p for transformation efficiency are indicated by *, p < 5% and **, p < 0.5%.
