# Supplementary figures and images for: Natural transformation of the filamentous cyanobacterium Phormidium lacuna
Source: PLoS One. 2020 Jun 12;15(6):e0234440. doi: 10.1371/journal.pone.0234440 (PMC7292380; doi:10.1371/journal.pone.0234440)

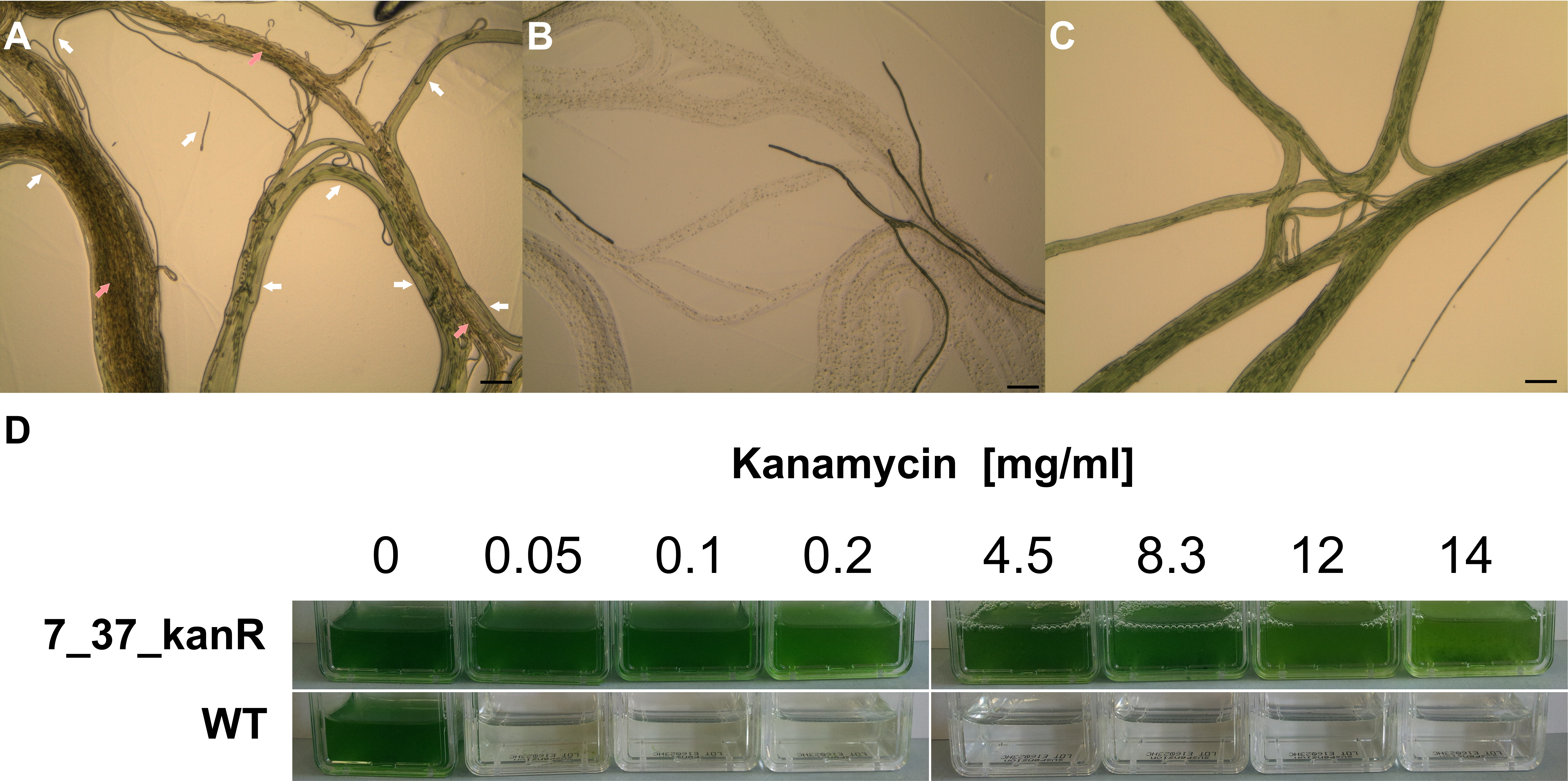

Supplement: S1 Original Images — (ZIP) [file pone.0234440.s002.zip › Fig 1.tif]

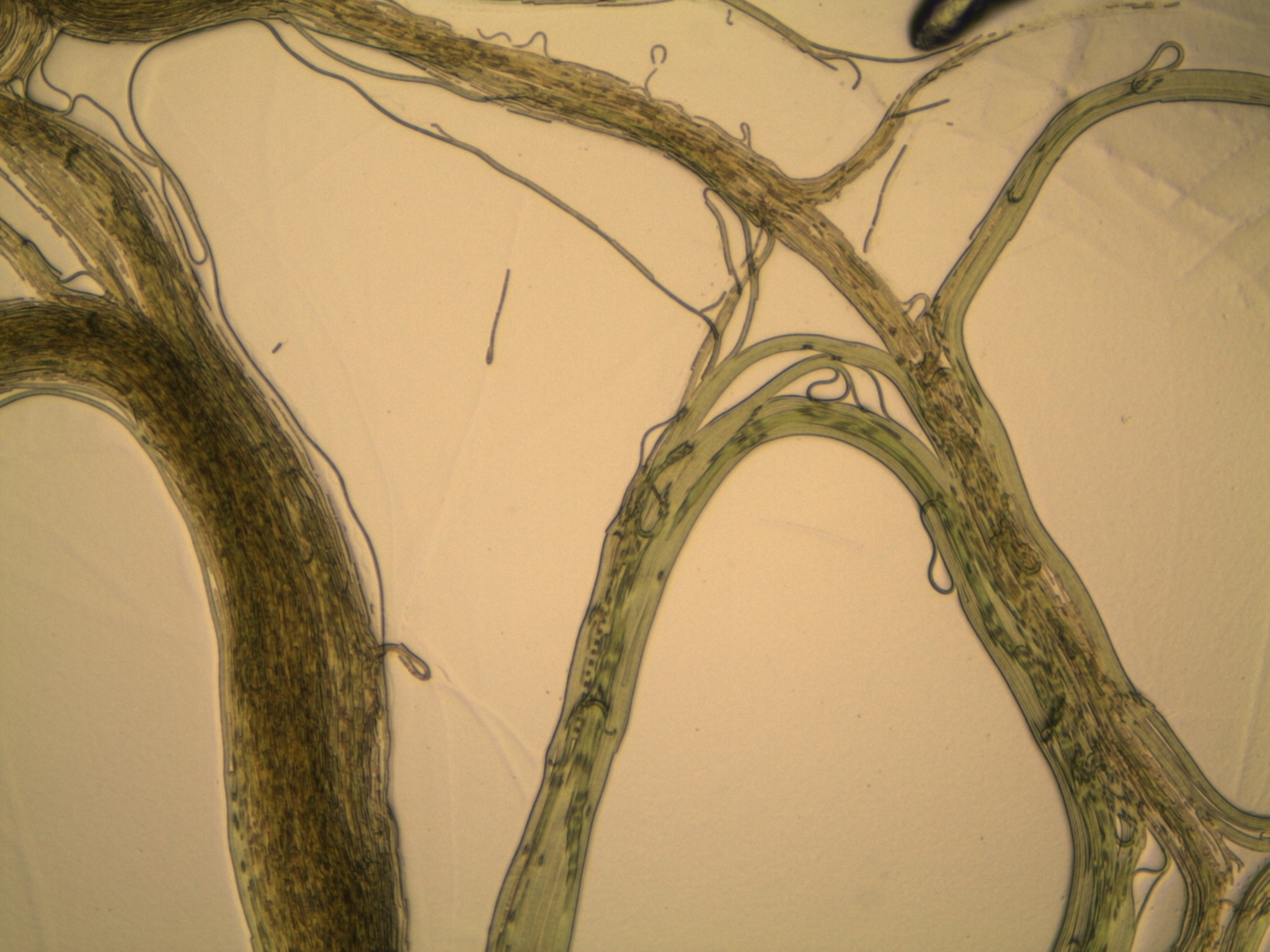

Supplement: S1 Original Images — (ZIP) [file pone.0234440.s002.zip › Figure 1A original.tif]

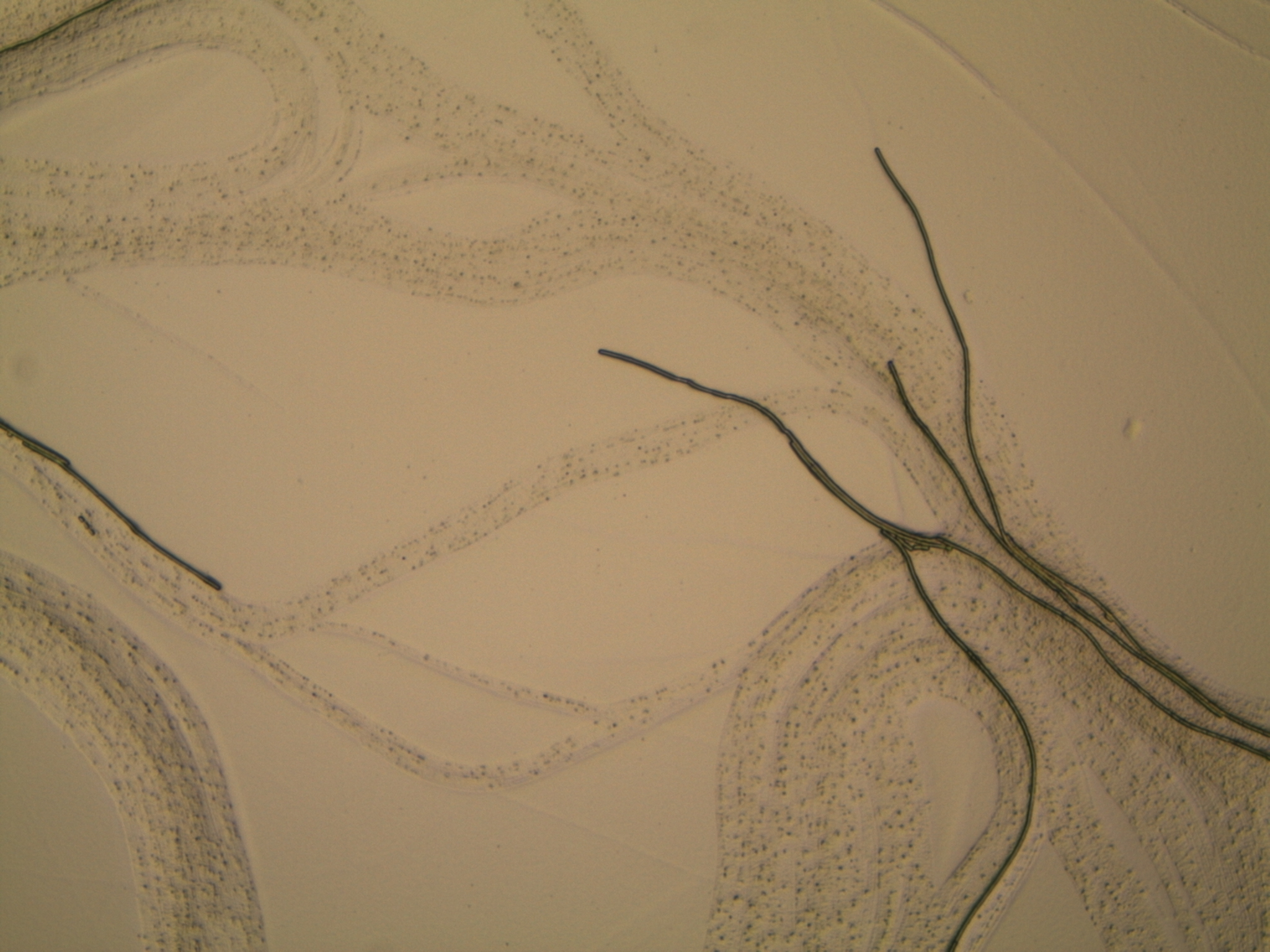

Supplement: S1 Original Images — (ZIP) [file pone.0234440.s002.zip › Figure 1B original.tif]

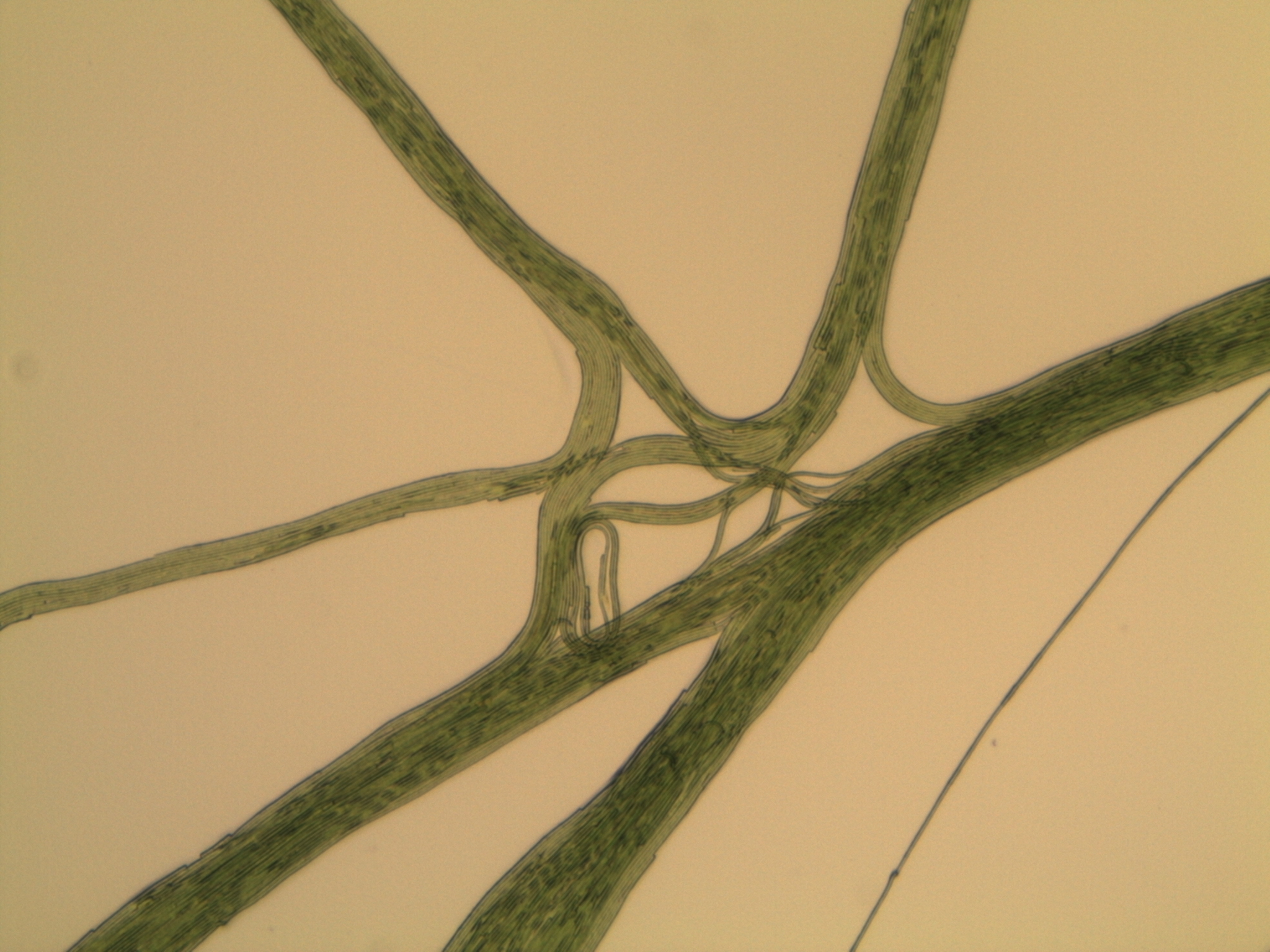

Supplement: S1 Original Images — (ZIP) [file pone.0234440.s002.zip › Figure 1C original.tif]

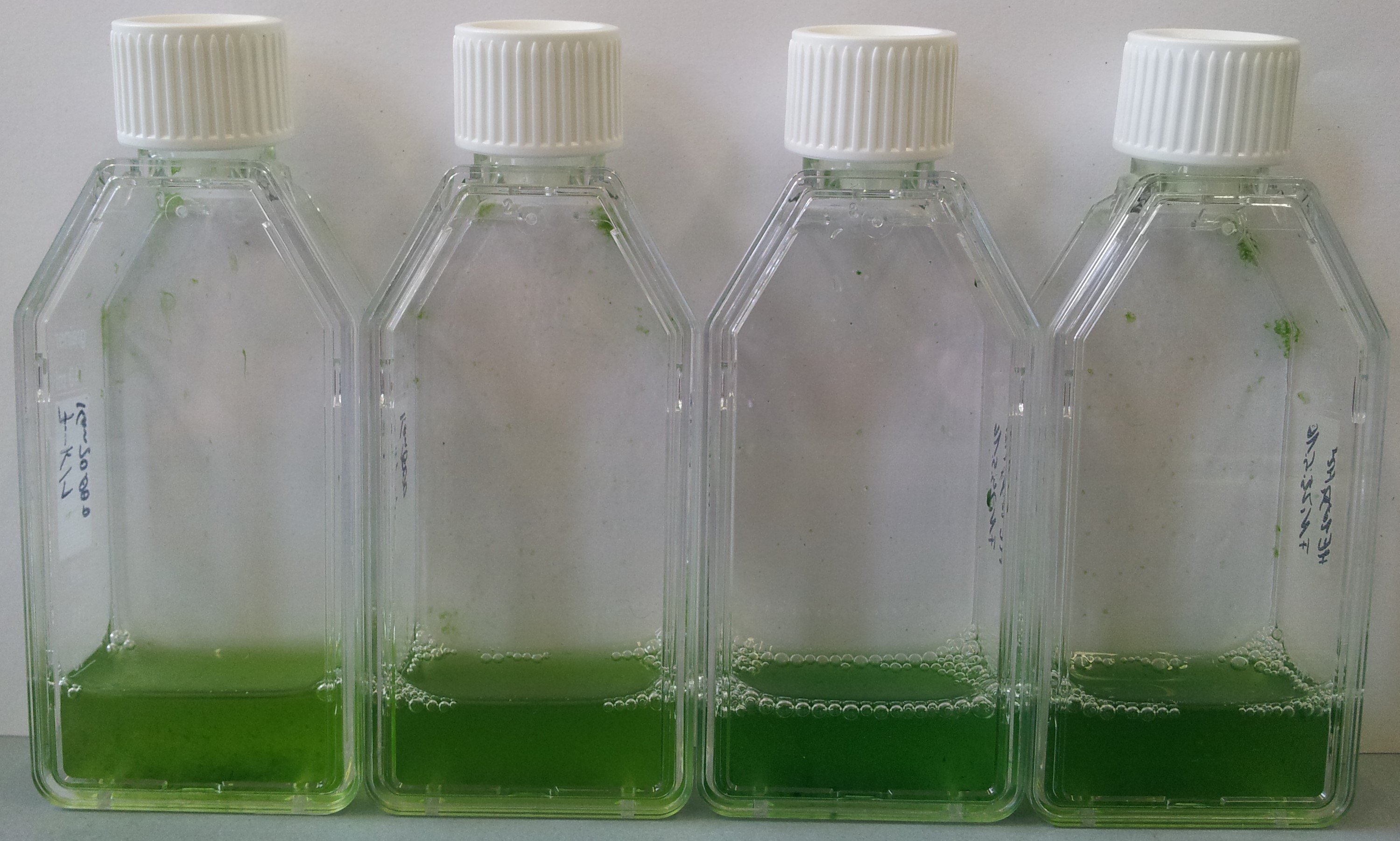

Supplement: S1 Original Images — (ZIP) [file pone.0234440.s002.zip › Figure 1D mutant high.jpg]

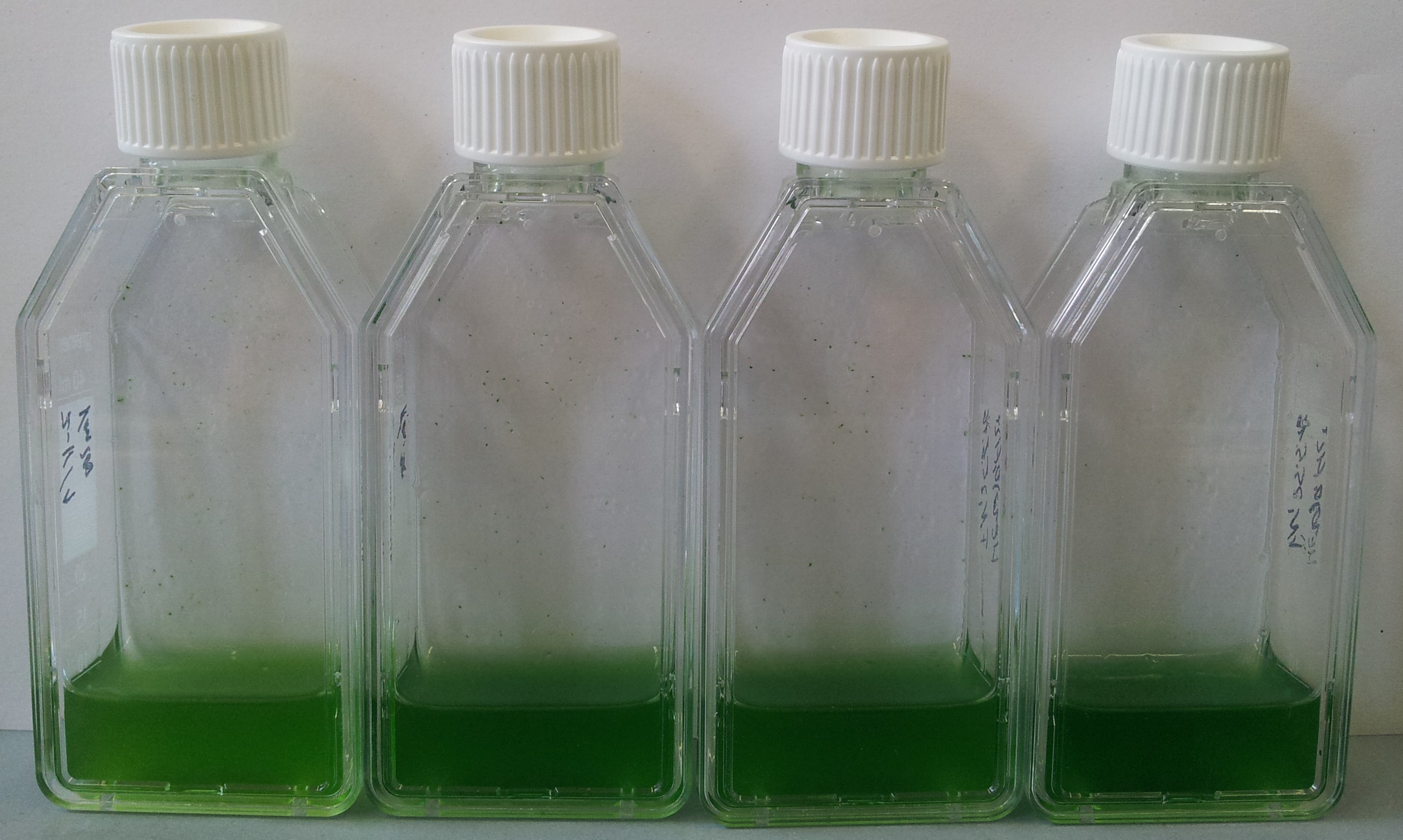

Supplement: S1 Original Images — (ZIP) [file pone.0234440.s002.zip › Figure 1D original mutant low.jpg]

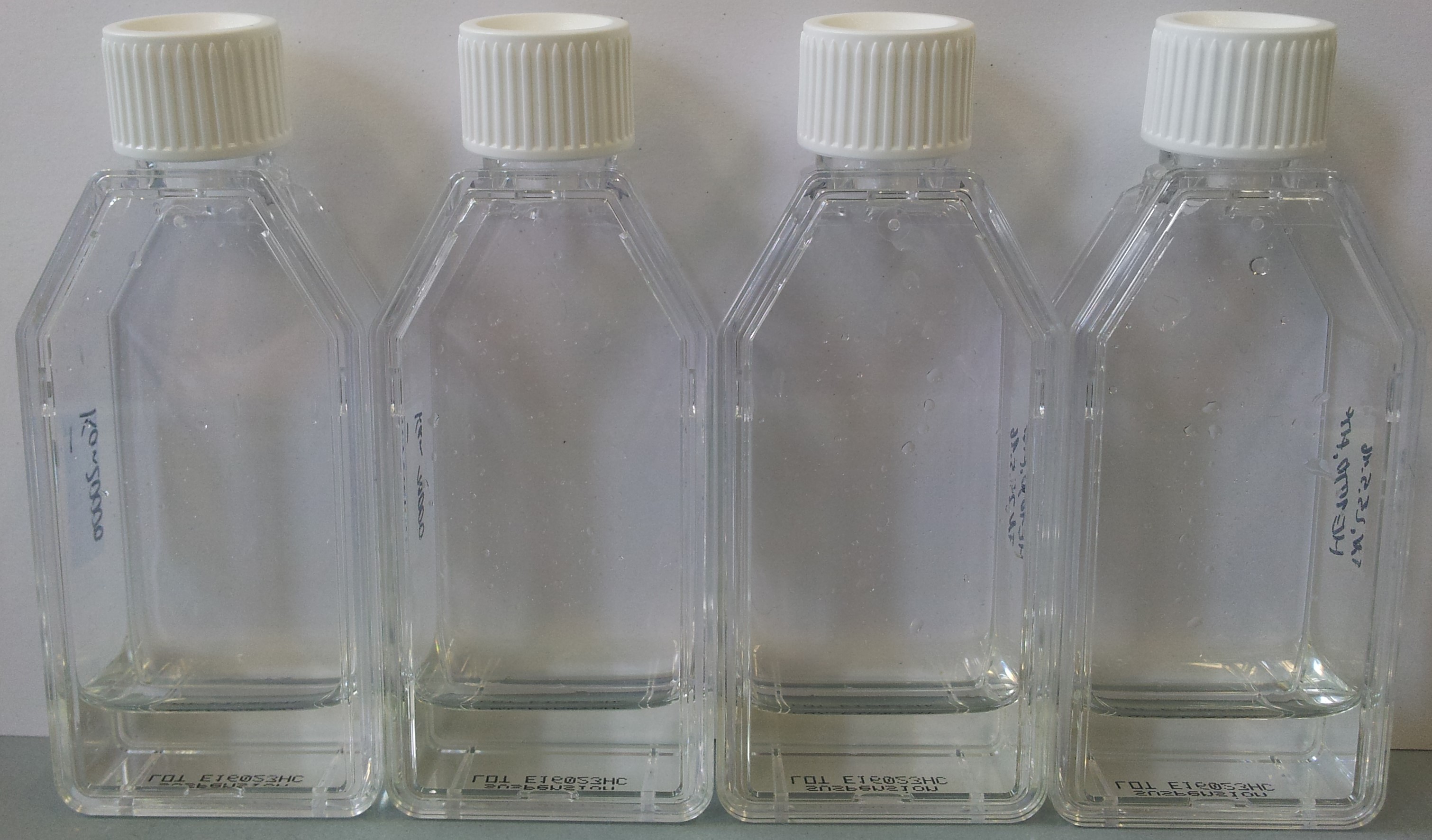

Supplement: S1 Original Images — (ZIP) [file pone.0234440.s002.zip › Figure 1D original wild type high.jpg]

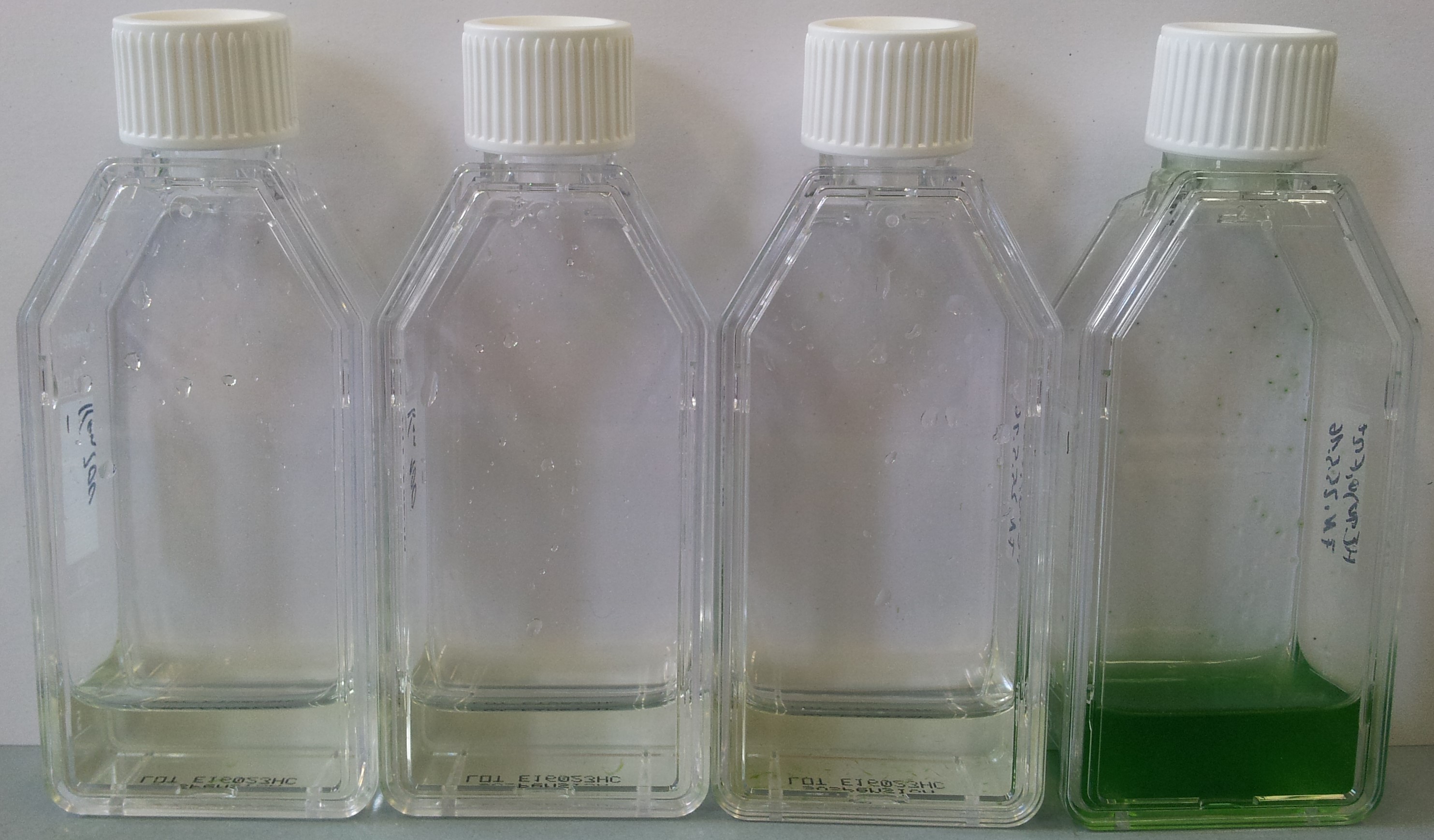

Supplement: S1 Original Images — (ZIP) [file pone.0234440.s002.zip › Figure 1D original wildtype low.jpg]

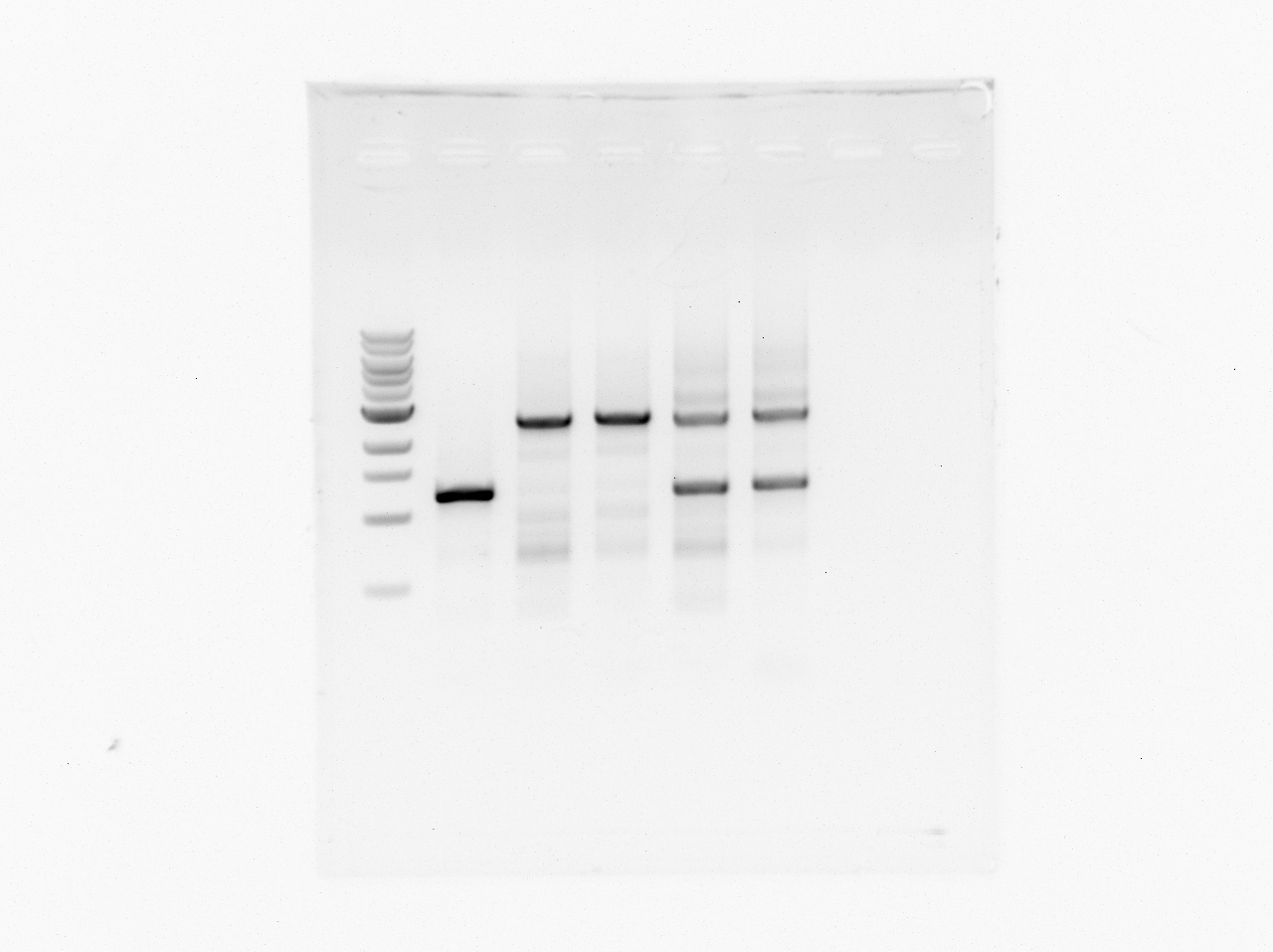

Supplement: S1 Original Images — (ZIP) [file pone.0234440.s002.zip › Figure 2C original.tif]

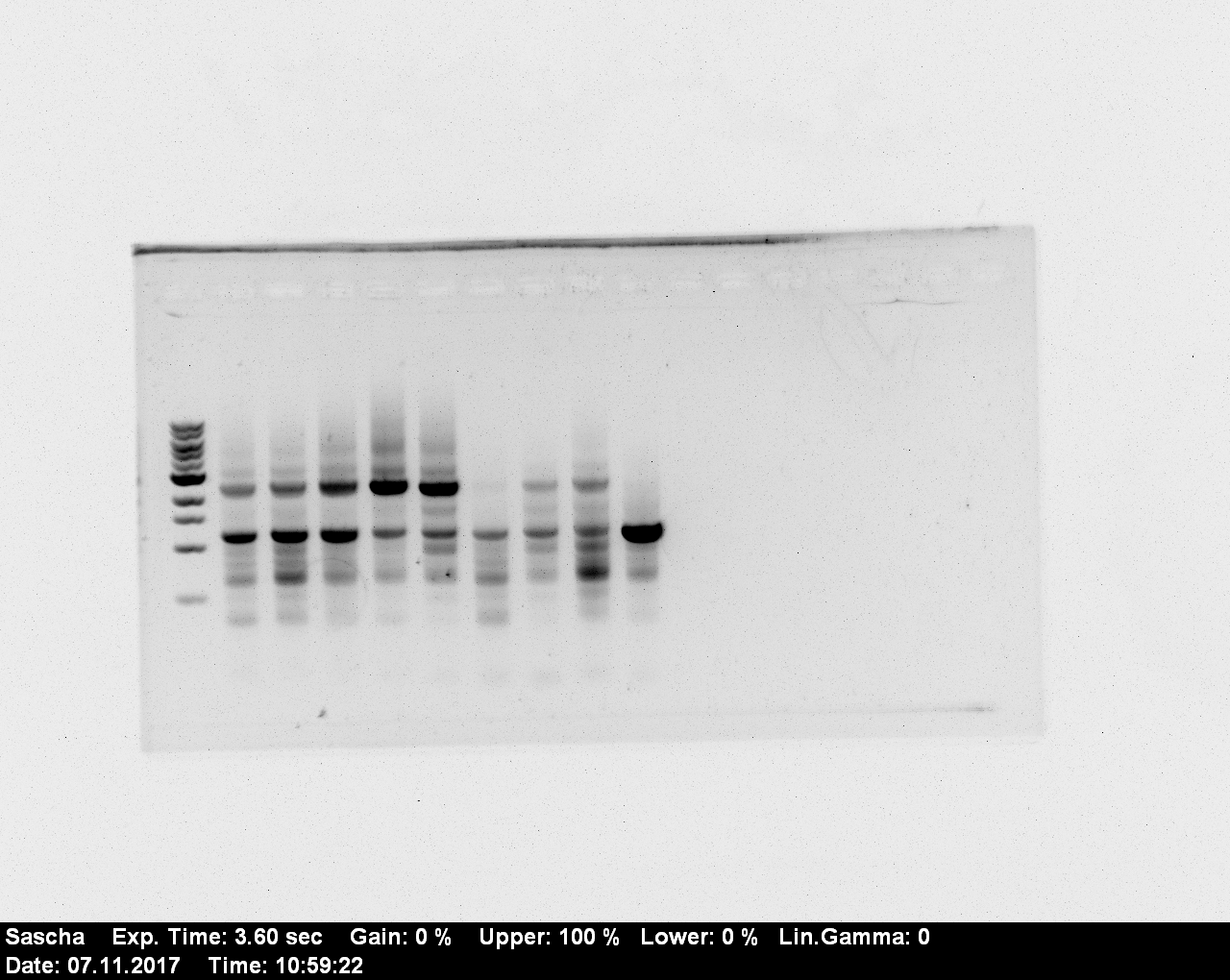

Supplement: S1 Original Images — (ZIP) [file pone.0234440.s002.zip › Figure 4A original.tif]

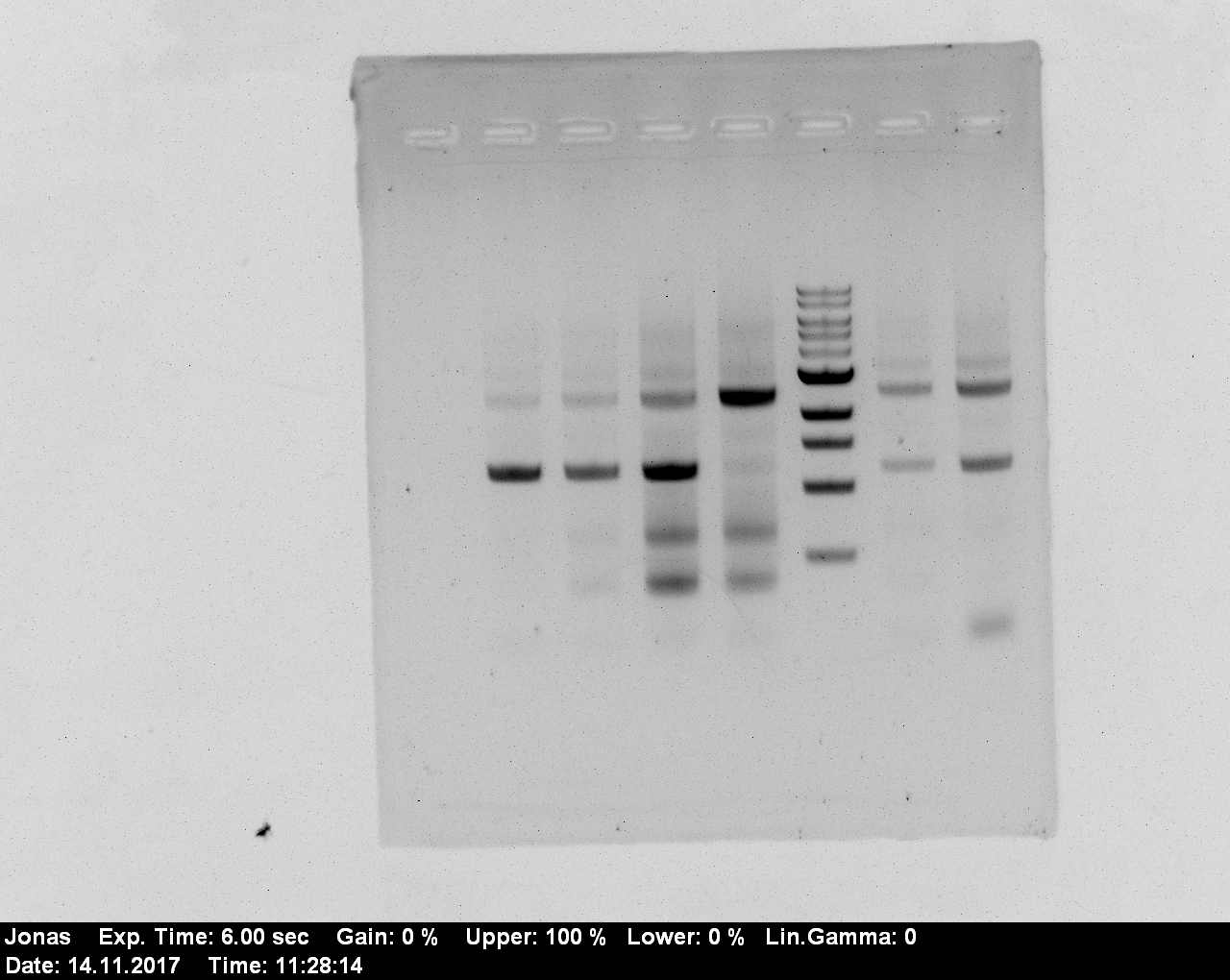

Supplement: S1 Original Images — (ZIP) [file pone.0234440.s002.zip › Figure 4B original.tif]

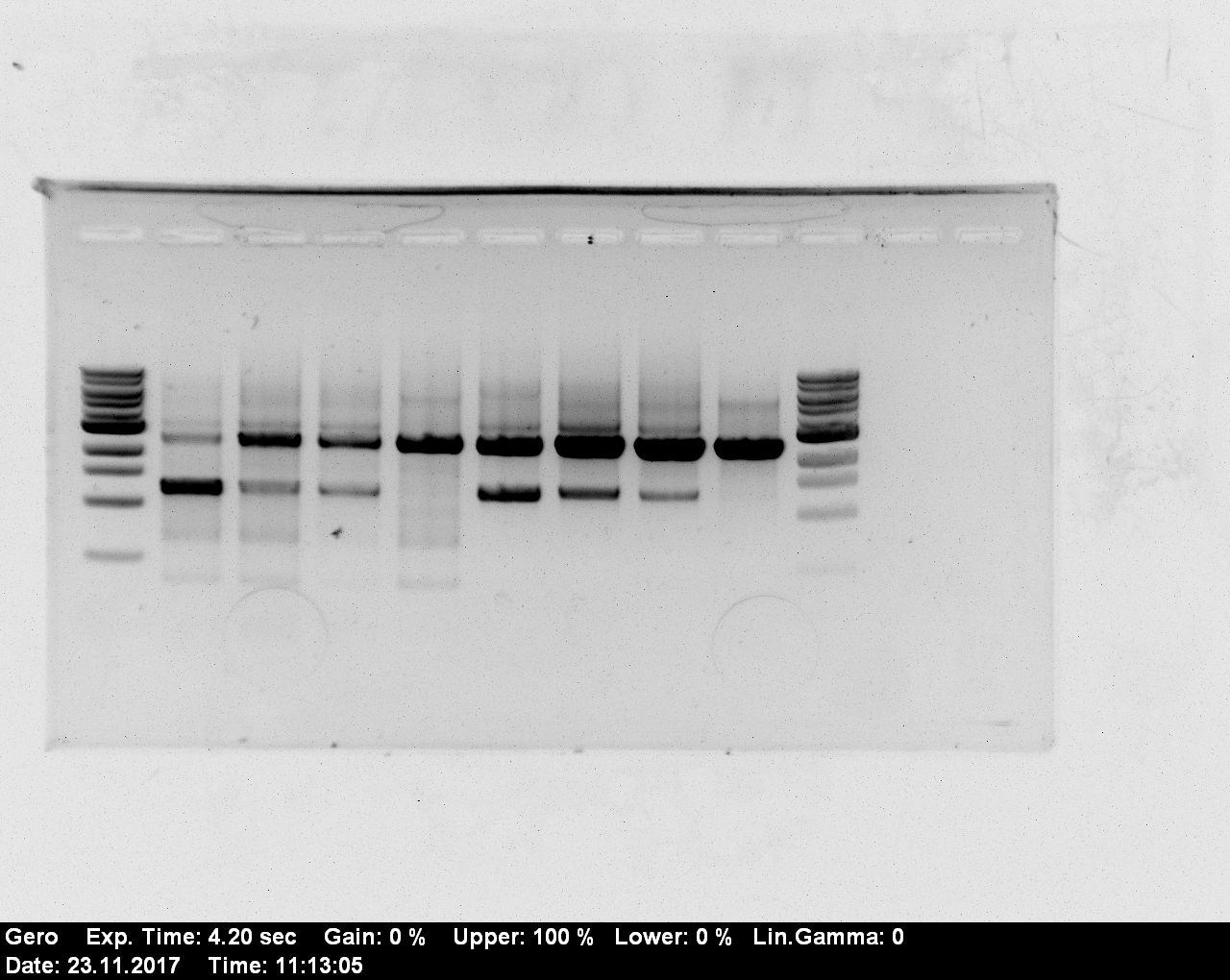

Supplement: S1 Original Images — (ZIP) [file pone.0234440.s002.zip › Figure 4C original.tif]

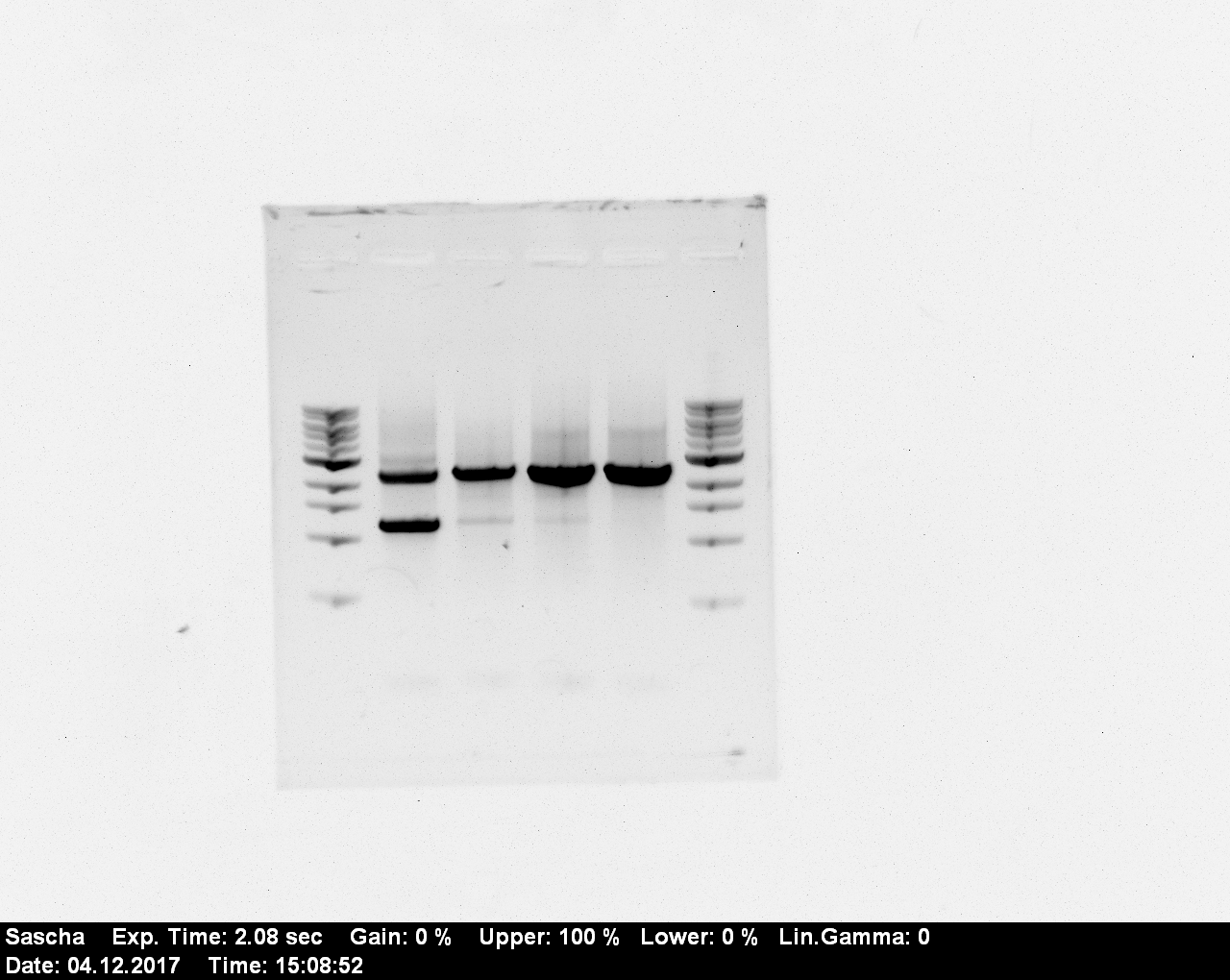

Supplement: S1 Original Images — (ZIP) [file pone.0234440.s002.zip › Figure 4D original.tif]

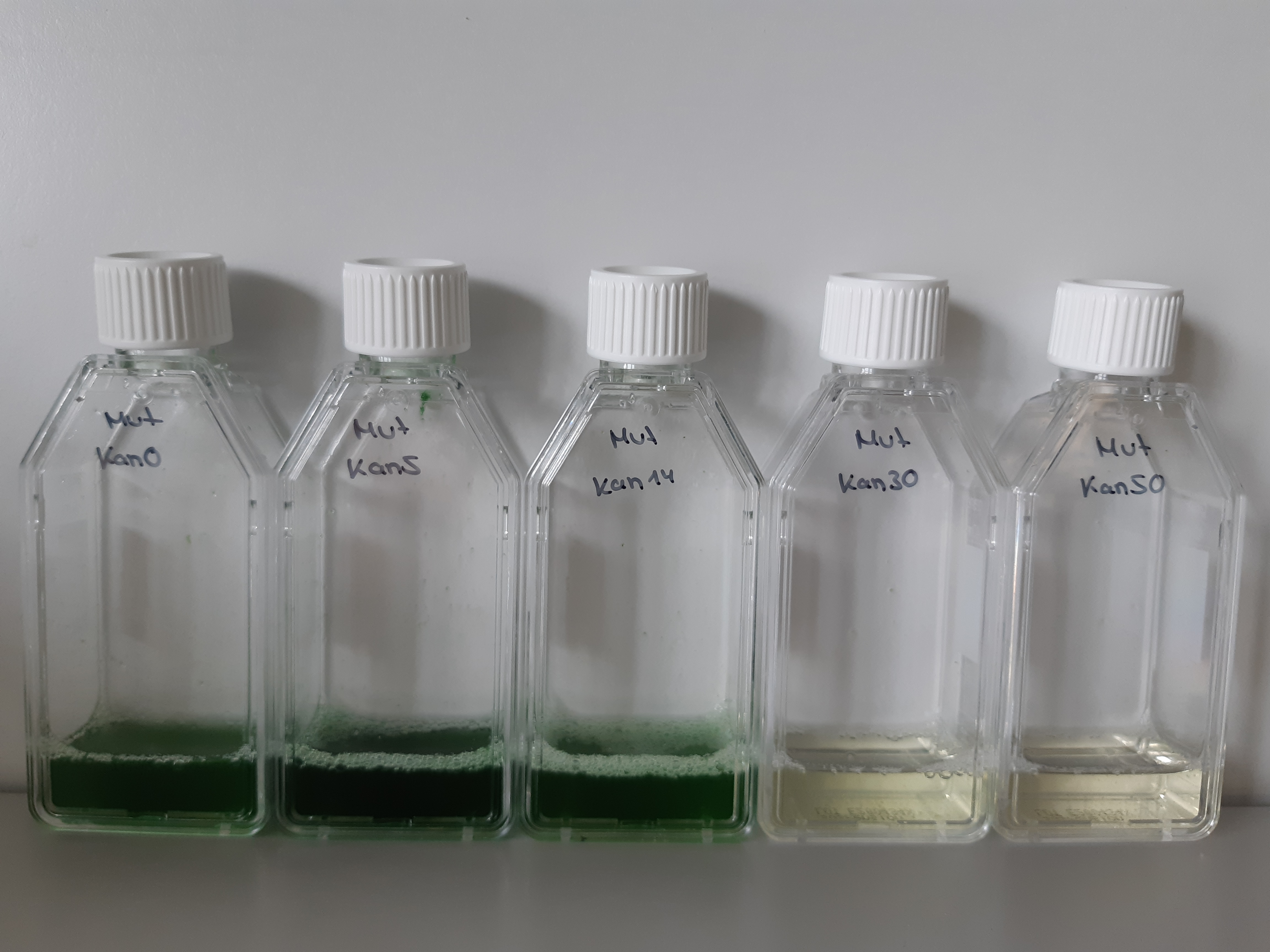

Supplement: S1 Original Images — (ZIP) [file pone.0234440.s002.zip › Figure S1 original .jpg]

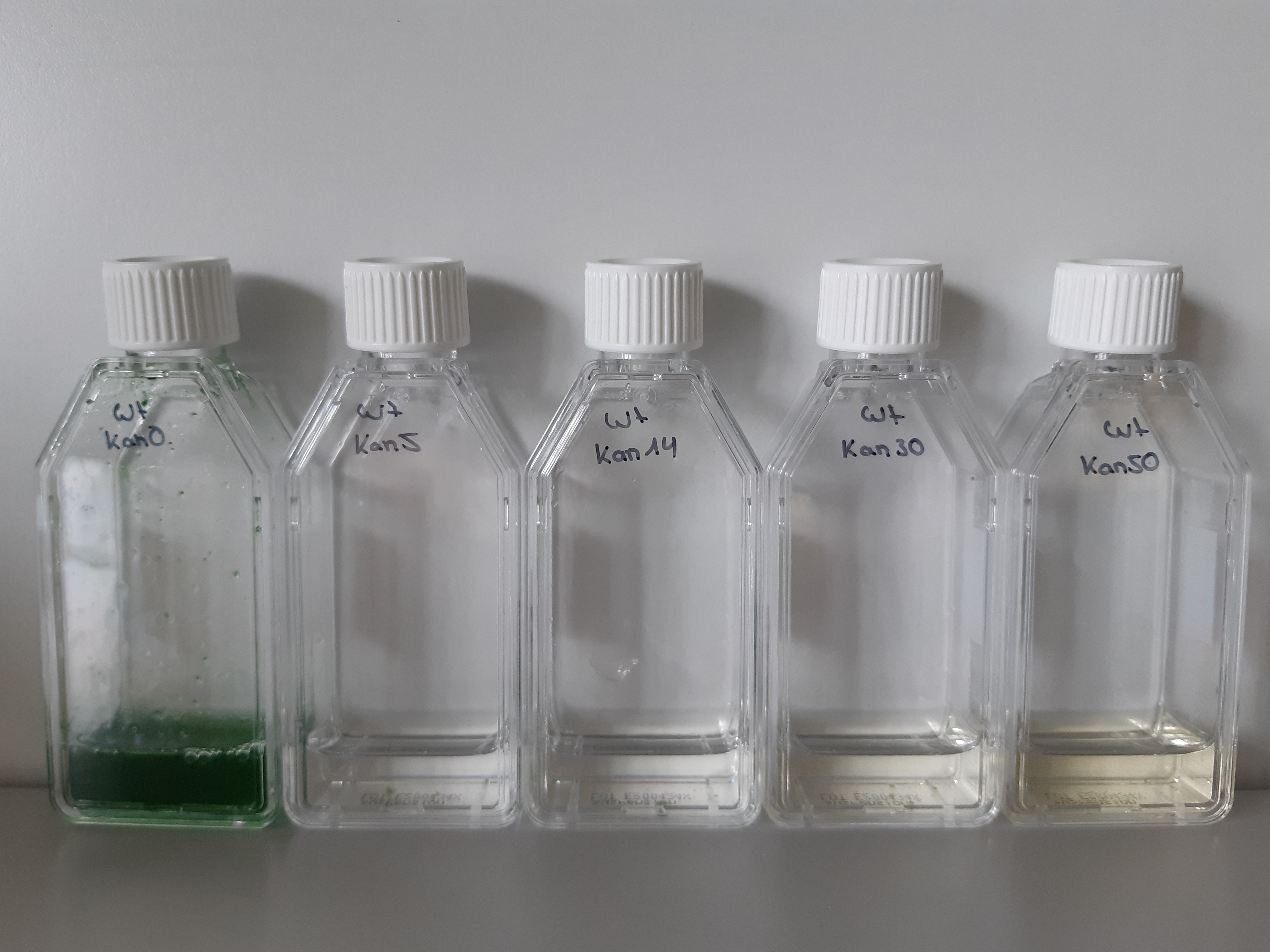

Supplement: S1 Original Images — (ZIP) [file pone.0234440.s002.zip › Figure s1 original wild type.jpg]
